# Supplementary material for: Community engagement in Indigenous food systems contamination studies: A systematic scoping review
Source: PLoS One. 2025 Nov 14;20(11):e0336439. doi: 10.1371/journal.pone.0336439 (PMC12617898; doi:10.1371/journal.pone.0336439)
Supplement: S1 Table — (DOCX) [file pone.0336439.s001.docx]

| **Authors** | **Title** | **Publication**  **Year** | **Journal** |
| --- | --- | --- | --- |
| Achatz, R.W.; de Vasconcellos, A.C.S.; Pereira, L.; Viana, P.V.S.; Basta, P.C. | Impacts of the goldmining and chronic methylmercury exposure on the good-living and mental health of munduruku native communities in the amazon basin | 2021 | Int. J. Environ. Res. Public health |
| Achouba, A.; Dumas, P.; Ouellet, N.; Lemire, M.; Ayotte, P. | Plasma levels of selenium-containing proteins in Inuit adults from Nunavik | 2016 | Environ. Int. |
| Adamou, T.Y.; Riva, M.; Muckle, G.; Laouan Sidi, E.A.; Lemire, M.; Ayotte, P. | Blood mercury and plasma polychlorinated biphenyls concentrations in pregnant Inuit women from Nunavik: Temporal trends, 1992–2017 | 2020 | Sci. Total environ. |
| Adamou, T.Y.; Riva, M.; Muckle, G.; Laouan-Sidi, E.A.; Ayotte, P. | Socio-economic inequalities in blood mercury (Hg) and serum polychlorinated biphenyl (PCB) concentrations among pregnant Inuit women from Nunavik, Canada | 2018 | Can. J. Public health |
| Adams, E.M.; von Hippel, F.A.; Hungate, B.A.; Buck, C.L. | Polychlorinated biphenyl (PCB) contamination of subsistence species on Unalaska Island in the Aleutian Archipelago | 2019 | Heliyon |
| Adhikari, S.; Siebert, S. J.; Jordaan, A. | Chromium dust deposition on Moringa oleifera leaves harvested by local communities in Sekhukhuneland, South Africa. | 2021 | Acta horticulturae |
| Akbar, L.; Zuk, A.M.; Martin, I.D.; Liberda, E.N.; Tsuji, L.J.S. | Potential obesogenic effect of a complex contaminant mixture on Cree First Nations adults of Northern Québec, Canada | 2021 | Environ. Res. |
| Aker, A.; Ayotte, P.; Caron-Beaudoin, E.; De Silva, A.; Ricard, S.; Gaudreau, É.; Lemire, M. | Plasma concentrations of perfluoroalkyl acids and their determinants in youth and adults from Nunavik, Canada | 2023 | Chemosphere |
| Aker, A.; Ayotte, P.; Caron-Beaudoin, É.; De Silva, A.; Ricard, S.; Lemire, M. | Associations between dietary profiles and perfluoroalkyl acids in Inuit youth and adults | 2023 | Sci. Total environ. |
| Aker, A.; Caron-Beaudoin, É.; Ayotte, P.; Ricard, S.; Gilbert, V.; Avard, E.; Lemire, M. | Non-persistent exposures from plasticizers or plastic constituents in remote Arctic communities: a case for further research | 2022 | J. Expos. Sci. Environ. Epidemiol. |
| Akwesasne Task Force Environm; Aminov, Z; Haase, R; Rej, R; Schymura, MJ; Santiago-Rivera, A; Morse, G; DeCaprio, A; Carpenter, DO | Diabetes Prevalence in Relation to Serum Concentrations of Polychlorinated Biphenyl (PCB) Congener Groups and Three Chlorinated Pesticides in a Native American Population | 2016 | Environmental health perspectives |
| Alcala-Orozco, M.; Caballero-Gallardo, K.; Olivero-Verbel, J. | Mercury exposure assessment in indigenous communities from Tarapaca village, Cotuhe and Putumayo Rivers, Colombian Amazon | 2019 | Environ. Sci. Pollut. Res. |
| Anbleyth-Evans, J.; Leiva, F.A.; Rios, F.T.; Cortés, R.S.; Vreni Häussermann; Aguirre-Munoz, C. | Toward marine democracy in Chile: Examining aquaculture ecological impacts through common property local ecological knowledge | 2020 | Mar. Policy |
| Andrade-Rivas, F.; Afshari, R.; Yassi, A.; Mardani, A.; Taft, S.; Guttmann, M.; Rao, A.S.; Thomas, S.; Takaro, T.; Spiegel, J.M. | Industrialization and food safety for the Tsleil-Waututh Nation: An analysis of chemical levels in shellfish in Burrard Inlet | 2022 | Environ. Res. |
| Anticona, C.; Bergdahl, I.A.; Sebastian, M.S. | Lead exposure among children from native communities of the Peruvian Amazon basin | 2012 | Rev. Panam. Salud publica pan am. J. Public health |
| Arrifano, GPF; Martin-Doimeadios, RCR; Jimenez-Moreno, M; Fernandez-Trujillo, S; Augusto-Oliveira, M; Souza-Monteiro, JR; Macchi, BM; Alvarez-Leite, JI; do Nascimento, JLM; Amador, MT; Santos, S; Ribeiro-dos-Santos, A; Silva-Pereira, LC; Oria, RB; Crespo-Lopez, ME | Genetic Susceptibility to Neurodegeneration in Amazon: Apolipoprotein E Genotyping in Vulnerable Populations Exposed to Mercury | 2018 | Frontiers in genetics |
| Baldwin, C; Bradford, L; Carr, MK; Doig, LE; Jardine, TD; Jones, PD; Bharadwaj, L; Lindenschmidt, KE | Ecological patterns of fish distribution in the Slave River Delta region, Northwest Territories, Canada, as relayed by traditional knowledge and Western science | 2018 | International journal of water resources development |
| Bank-Nielsen, P.I.; Long, M.; Bonefeld-Jørgensen, E.C. | Pregnant inuit women’s exposure to metals and association with fetal growth outcomes: ACCEPT 2010–2015 | 2019 | Int. J. Environ. Res. Public health |
| Basta, P.C.; De Sousa Viana, P.V.; De Vasconcellos, A.C.S.; Santos Périssé, A.R.; Hofer, C.B.; Paiva, N.S.; Kempton, J.W.; De Andrade, D.C.; De Oliveira, R.A.A.; Achatz, R.; Perini, J.A.; De Moura Meneses, H.D.N.; Hallwass, G.; De Oliveira Lima, M.; Maura De Jesus, I.; Ribeiro Dos Santos, C.C.; De Souza Hacon, S. | Mercury exposure in munduruku indigenous communities from brazilian amazon: Methodological background and an overview of the principal results | 2021 | Int. J. Environ. Res. Public health |
| Benefice, E.; Luna-Monrroy, S.; Lopez-Rodriguez, R. | Fishing activity, health characteristics and mercury exposure of Amerindian women living alongside the Beni River (Amazonian Bolivia) | 2010 | Int. J. Hyg. Environ. Health |
| Berky, A.J.; Robie, E.; Chipa, S.N.; Ortiz, E.J.; Palmer, E.J.; Rivera, N.A.; Avalos, A.M.M.; Meyer, J.N.; Hsu-Kim, H.; Pan, W.K. | Risk of lead exposure from wild game consumption from cross-sectional studies in Madre de Dios, Peru | 2022 | Lancet reg. Health - am. |
| Bhattacharya, B.; Chandra Deka, D. | Metal Contents in Traditional Alcoholic Rice Beers Prepared by Rabha and Sonowal Kachari Tribes of Assam, India | 2023 | Asian j. Chem. |
| Binnington, M.J.; Curren, M.S.; Chan, H.M.; Wania, F. | Balancing the benefits and costs of traditional food substitution by indigenous Arctic women of childbearing age: Impacts on persistent organic pollutant, mercury, and nutrient intakes | 2016 | Environ. Int. |
| Binnington, M.J.; Curren, M.S.; Quinn, C.L.; Armitage, J.M.; Arnot, J.A.; Chan, H.M.; Wania, F. | Mechanistic polychlorinated biphenyl exposure modeling of mothers in the Canadian Arctic: The challenge of reliably establishing dietary composition | 2016 | Environ. Int. |
| Binnington, MJ; Lei, YD; Pokiak, L; Pokiak, J; Ostertag, SK; Loseto, LL; Chan, HM; Yeung, LWY; Huang, HY; Wania, F | Effects of preparation on nutrient and environmental contaminant levels in Arctic beluga whale (Delphinapterus leucas) traditional foods | 2017 | Environmental science-processes & impacts |
| Blanco, GD; Hanazaki, N; Cunha, SMB; Cremer, MJ; Campos, ML | Exploring the Interfaces between Ethnobiology and Ecotoxicology: A Novel Approach | 2020 | Ethnobiology letters |
| Bordeleau, S.; Asselin, H.; Mazerolle, M.J.; Imbeau, L. | "Is it still safe to eat traditional food?" Addressing traditional food safety concerns in aboriginal communities | 2016 | Sci. Total environ. |
| Boucher, O.; Muckle, G.; Ayotte, P.; Dewailly, E.; Jacobson, S.W.; Jacobson, J.L. | Altered fine motor function at school age in Inuit children exposed to PCBs, methylmercury, and lead | 2016 | Environ. Int. |
| Brunet, N.D.; Jardine, T.D.; Jones, P.D.; Macdermid, F.; Reed, G.; Bogdan, A.-M.; Tchir, D.R.; Natcher, D.C. | Towards indigenous community-led monitoring of fish in the oil sands region of Canada: Lessons at the intersection of cultural consensus and fish science | 2020 | Extr. Ind. Soc. |
| Byrne, S; Seguinot-Medina, S; Miller, P; Waghiyi, V; von Hippel, FA; Buck, CL; Carpenter, DO | Exposure to polybrominated diphenyl ethers and perfluoroalkyl substances in a remote population of Alaska Natives | 2017 | Environmental pollution |
| Byrne, S.; Seguinot-Medina, S.; Waghiyi, V.; Apatiki, E.; Immingan, T.; Miller, P.; von Hippel, F.A.; Buck, C.L.; Carpenter, D.O. | PFAS and PBDEs in traditional subsistence foods from Sivuqaq, Alaska | 2022 | Environ. Sci. Pollut. Res. |
| Byrne, S.C.; Miller, P.; Seguinot-Medina, S.; Waghiyi, V.; Buck, C.L.; von Hippel, F.A.; Carpenter, D.O. | Exposure to perfluoroalkyl substances and associations with serum thyroid hormones in a remote population of Alaska Natives | 2018 | Environ. Res. |
| Calder, R.S.D.; Bromage, S.; Sunderland, E.M. | Risk tradeoffs associated with traditional food advisories for Labrador Inuit | 2019 | Environ. Res. |
| Caron-Beaudoin, É.; Armstrong, C.G. | Biomonitoring and Ethnobiology: Approaches to Fill Gaps in Indigenous Public and Environmental Health | 2019 | J. Ethnobiology |
| Chaisson, A.M.; Franklin, C.A.; Zender, L.; Chaisson, C.F.; Sheldon, R.; Foran, J.A. | A community-based application of software to conduct a probabilistic assessment of exposure to contaminants in indigenous subsistence foods | 2012 | Environ. Justice |
| Champeau, O; Ataria, JM; Northcott, GL; Kume, G; Barrick, A; Tremblay, LA | Assessment of the Impacts of Anthropogenic Activities on a Large River Using Longfin Eel as a Bioindicator | 2020 | Sustainability |
| Charania, N.A.; Tsuji, L.J.S.; Martin, I.D.; Liberda, E.N.; Coté, S.; Ayotte, P.; Dewailly, E.; Nieboer, E. | An examination of traditional foods and cigarette smoking as cadmium sources among the nine First Nations of Eeyou Istchee, northern Quebec, Canada | 2014 | Environ. Sci. Process. Impacts |
| Charette, T.; Kaminski, G.; Rosabal, M.; Amyot, M. | Effects of speciation, cooking and changes in bioaccessibility on methylmercury exposure assessment for contrasting diets of fish and marine mammals | 2021 | Int. J. Environ. Res. Public health |
| Chashchin, V.; Kovshov, A.A.; Thomassen, Y.; Sorokina, T.; Gorbanev, S.A.; Morgunov, B.; Gudkov, A.B.; Chashchin, M.; Sturlis, N.V.; Trofimova, A.; Odland, J.Ø.; Nieboer, E. | Health risk modifiers of exposure to persistent pollutants among indigenous peoples of Chukotka | 2020 | Int. J. Environ. Res. Public health |
| Cooke, M.W.; Trudel, M.; Gurney-Smith, H.J.; Kellogg, J.P.; Cullen, J.T.; Francisco, B.B.A.; Mercier, J.F.; Chen, J. | Radioactivity concentration measurements in fish and shellfish samples from the west coast of Canada after the Fukushima nuclear accident (2011–2018) | 2022 | J. Environ. Radioact. |
| Cordier, S.; Anassour-Laouan-Sidi, E.; Lemire, M.; Costet, N.; Lucas, M.; Ayotte, P. | Association between exposure to persistent organic pollutants and mercury, and glucose metabolism in two Canadian Indigenous populations | 2020 | Environ. Res. |
| Cott, PA; Amos, AL; Guzzo, MM; Chavarie, L; Goater, CP; Muir, DCG; Evans, MS | Can traditional methods of selecting food accurately assess fish health? | 2018 | Arctic science |
| Curren, MS; Davis, K; Liang, CL; Adlard, B; Foster, WG; Donaldson, SG; Kandola, K; Brewster, J; Potyrala, M; Van Oostdam, J | Comparing plasma concentrations of persistent organic pollutants and metals in primiparous women from northern and southern Canada | 2014 | Science of the total environment |
| Curren, MS; Liang, CL; Davis, K; Kandola, K; Brewster, J; Potyrala, M; Chan, HM | Assessing determinants of maternal blood concentrations for persistent organic pollutants and metals in the eastern and western Canadian Arctic | 2015 | Science of the total environment |
| da Silva, L.V.D.; Pereira, S.F.P.; Carneiro, C.C.; e Silva, T.M.; Rocha, R.M.; da Costa, H.C.; e Silva, C.S.; de Souza, A.M.F.; da Silva, M.L.S. | Assessment of the Health Risk of Indigenous People by the Consumption of Fish with Hg and As in Villages Located Close to Mining | 2023 | J. Braz. Chem. Soc. |
| de Bakker, L.B.; Gasparinetti, P.; de Queiroz, J.M.; de Vasconcellos, A.C.S. | Economic impacts on human health resulting from the use of mercury in the illegal gold mining in the brazilian amazon: A methodological assessment | 2021 | Int. J. Environ. Res. Public health |
| De Loma, J.; Tirado, N.; Ascui, F.; Levi, M.; Vahter, M.; Broberg, K.; Gardon, J. | Elevated arsenic exposure and efficient arsenic metabolism in indigenous women around Lake Poopó Bolivia | 2019 | Sci. Total environ. |
| de Matos, L.S.; Silva, J.O.S.; Kasper, D.; Carvalho, L.N. | Assessment of mercury contamination in Brycon falcatus (Characiformes: Bryconidae) and human health risk by consumption of this fish from the Teles Pires River, Southern Amazonia | 2018 | Neotrop. Ichthyol. |
| de Oliveira, D.F.; de Castro, B.S.; do Nascimento Recktenvald, M.C.N.; da Costa Júnior, W.A.; da Silva, F.X.; de Menezes Alves, C.L.; Froehlich, J.D.; Bastos, W.R.; Ott, A.M.T. | Mercury in wild animals and fish and health risk for indigenous Amazonians | 2021 | Food. Addit. Contam. Part b surveill. |
| de Vasconcellos, A.C.S.; Hallwass, G.; Bezerra, J.G.; Aciole, A.N.S.; Meneses, H.N.M.; Lima, M.O.; de Jesus, I.M.; Hacon, S.S.; Basta, P.C. | Health risk assessment of mercury exposure from fish consumption in munduruku indigenous communities in the brazilian amazon | 2021 | Int. J. Environ. Res. Public health |
| Deere, J.R.; Jankowski, M.D.; Primus, A.; Phelps, N.B.D.; Ferrey, M.; Borucinska, J.; Chenaux-Ibrahim, Y.; Isaac, E.J.; Singer, R.S.; Travis, D.A.; Moore, S.; Wolf, T.M. | Health of wild fish exposed to contaminants of emerging concern in freshwater ecosystems utilized by a Minnesota Tribal community | 2024 | Integr. Environ. assess. manage. |
| Deere, J.R.; Moore, S.; Ferrey, M.; Jankowski, M.D.; Primus, A.; Convertino, M.; Servadio, J.L.; Phelps, N.B.D.; Hamilton, M.C.; Chenaux-Ibrahim, Y.; Travis, D.A.; Wolf, T.M. | Occurrence of contaminants of emerging concern in aquatic ecosystems utilized by Minnesota tribal communities | 2020 | Sci. Total environ. |
| Dellinger, M.J.; Anguzu, R.; Pingatore, N.; Ripley, M. | Risk-benefit modeling to guide health research in collaboration with Great Lakes fish consuming Native American communities | 2020 | J. Great lakes res. |
| Dellinger, M.J.; Lyons, M.; Clark, R.; Olson, J.; Pingatore, N.; Ripley, M. | Culturally adapted mobile technology improves environmental health literacy in Laurentian, Great Lakes Native Americans (Anishinaabeg) | 2019 | J. Great lakes res. |
| Dellinger, M.J.; Olson, J.; Clark, R.; Pingatore, N.; Ripley, M.P. | Development and pilot testing of a model to translate risk assessment data for Great Lakes Native American communities using mobile technology | 2018 | Hum. Ecol. Risk assess. |
| Dellinger, M.J.; Olson, J.T.; Holub, B.J.; Ripley, M.P. | Mercury, Polychlorinated Biphenyls, Selenium, and Fatty Acids in Tribal Fish Harvests of the Upper Great Lakes | 2018 | Risk anal. |
| Dellinger, M.J.; Pingatore, N.; Chelius, T.; Visotcky, A.; Sparapani, R.; Ripley, M. | Environmental health literacy for Anishinaabe (Great Lakes Native American) fish consumers: A randomized control trial | 2022 | Environ. Res. |
| Dellinger, M.J.; Ripley, M.P. | Mercury risks versus nutritional benefits of tribal commercial fish harvests in the Upper Laurentian Great Lakes | 2016 | Hum. Ecol. Risk assess. |
| Diringer, S.E.; Feingold, B.J.; Ortiz, E.J.; Gallis, J.A.; Araújo-Flores, J.M.; Berky, A.; Pan, W.K.Y.; Hsu-Kim, H. | River transport of mercury from artisanal and small-scale gold mining and risks for dietary mercury exposure in Madre de Dios, Peru | 2015 | Environ. Sci. Process. Impacts |
| Doyle, J.R.; Blais, J.M.; Holmes, R.D.; White, P.A. | A soil ingestion pilot study of a population following a traditional lifestyle typical of rural or wilderness areas | 2012 | Sci. Total environ. |
| Doyle, J.R.; Blais, J.M.; White, P.A. | A survey of the traditional food consumption that may contribute to enhanced soil ingestion in a Canadian First Nation community | 2012 | Sci. Total environ. |
| Drescher, O.; Dewailly, E.; Diorio, C.; Ouellet, N.; Sidi, E.A.L.; Abdous, B.; Valera, B.; Ayotte, P. | Methylmercury exposure, PON1 gene variants and serum paraoxonase activity in Eastern James Bay Cree adults | 2014 | J. Expos. Sci. Environ. Epidemiol. |
| Driscoll, D.; Sorensen, A.; Deerhake, M. | A multidisciplinary approach to promoting healthy subsistence fish consumption in culturally distinct communities | 2012 | Health promot. Pract. |
| Drysdale, M.; Gamberg, M.; Brammer, J.; Majowicz, S.E.; Packull-McCormick, S.; Skinner, K.; Laird, B.D. | Hexachlorobenzene and omega-3 fatty acid intake from traditional foods in the northern Yukon: A risk and benefit analysis | 2024 | Sci. Total environ. |
| Drysdale, M.; Ratelle, M.; Majowicz, S.E.; Brammer, J.; Gamberg, M.; Skinner, K.; Laird, B. | Traditional Food Consumption and Other Determinants of Exposure for Lead, Cobalt, Manganese, and Hexachlorobenzene in Northern Canada | 2023 | Arctic |
| Drysdale, M.; Ratelle, M.; Skinner, K.; Garcia-Barrios, J.; Gamberg, M.; Williams, M.; Majowicz, S.; Bouchard, M.; Stark, K.; Chalil, D.; Laird, B.D. | Human biomonitoring results of contaminant and nutrient biomarkers in Old Crow, Yukon, Canada | 2021 | Sci. Total environ. |
| Dudarev, A.A.; Yamin-Pasternak, S.; Pasternak, I.; Chupakhin, V.S. | Traditional diet and environmental contaminants in coastal chukotka IV: Recommended intake criteria | 2019 | Int. J. Environ. Res. Public health |
| Dudarev, AA; Chupakhin, VS; Odland, JO; Reiersen, LO; Chashchin, VP | A Follow-Up Study Of Blood Levels Of Persistent Toxic Substances (Pts) Among Indigenous Peoples Of Coastal Chukotka, Russia, 2001-2007 | 2010 | International journal of circumpolar health |
| El-Din Bekhit, A.; Al-Amer, S.; Gooneratne, R.; Mason, S.L.; Osman, K.A.; Clucas, L. | Concentrations of trace elementals and organochlorines in Mutton bird (Puffinus griseus) | 2011 | Ecotoxicol. Environ. Saf. |
| Forsberg, N.D.; Stone, D.; Harding, A.; Harper, B.; Harris, S.; Matzke, M.M.; Cardenas, A.; Waters, K.M.; Anderson, K.A. | Effect of native American fish smoking methods on dietary exposure to polycyclic aromatic hydrocarbons and possible risks to human health | 2012 | J. Agric. Food chem. |
| Furgal, C.M.; Boyd, A.D.; Mayeda, A.M.; Jardine, C.G.; Driedger, S.M. | Risk communication and perceptions about lead ammunition and Inuit health in Nunavik, Canada | 2023 | Int. J. Circumpolar health |
| Gadamus, L. | Linkages between human health and ocean health: A participatory climate change vulnerability assessment for marine mammal harvesters | 2013 | Int. J. Circumpolar health |
| Gallo, M.V.; Schell, L.M.; DeCaprio, A.P.; Jacobs, A. | Levels of persistent organic pollutant and their predictors among young adults | 2011 | Chemosphere |
| Gao, T.; Erokhin, V. | Capturing a complexity of nutritional, environmental, and economic impacts on selected health parameters in the Russian high north | 2020 | Sustainability |
| Garcia-Barrios, J.; Drysdale, M.; Ratelle, M.; Gaudreau, É.; LeBlanc, A.; Gamberg, M.; Laird, B.D. | Biomarkers of poly- and perfluoroalkyl substances (PFAS) in Sub-Arctic and Arctic communities in Canada | 2021 | Int. J. Hyg. Environ. Health |
| Garry, M.R.; Shock, S.S.; Salatas, J.; Dau, J. | Application of a weight of evidence approach to evaluating risks associated with subsistence caribou consumption near a lead/zinc mine | 2018 | Sci. Total environ. |
| Gaudin, V.L.; Receveur, O.; Walz, L.; Girard, F.; Potvin, L. | A mixed methods inquiry into the determinants of traditional food consumption among three Cree communities of Eeyou Istchee from an ecological perspective | 2014 | Int j circumpolar health |
| Golzadeh, N; Barst, BD; Basu, N; Baker, JM; Auger, JC; McKinney, MA | Evaluating the concentrations of total mercury, methylmercury, selenium, and selenium:mercury molar ratios in traditional foods of the Bigstone Cree in Alberta, Canada | 2020 | Chemosphere |
| Golzadeh, N.; Barst, B.D.; Baker, J.M.; Auger, J.C.; McKinney, M.A. | Alkylated polycyclic aromatic hydrocarbons are the largest contributor to polycyclic aromatic compound concentrations in traditional foods of the Bigstone Cree Nation in Alberta, Canada | 2021 | Environ. Pollut. |
| Guidotti, T.L. | Evaluating risk after a hazardous waste treatment plant released persistent organic pollutants. Part 2. Ecotoxicology and human health risk | 2018 | Case. Stud. Environ. |
| Guidotti, T.L. | Evaluating risk after a hazardous waste treatment plant released persistent organic pollutants: Part 3, Aboriginal health risk and impact | 2018 | Case. Stud. Environ. |
| Hacon, S.S.; Oliveira-Da-costa, M.; Gama, C.S.; Ferreira, R.; Basta, P.C.; Schramm, A.; Yokota, D. | Mercury exposure through fish consumption in traditional communities in the Brazilian Northern Amazon | 2020 | Int. J. Environ. Res. Public health |
| Hahn, J.L.; Van Alstyne, K.L.; Gaydos, J.K.; Wallis, L.K.; West, J.E.; Hollenhorst, S.J.; Ylitalo, G.M.; Poppenga, R.H.; Bolton, J.L.; McBride, D.E.; Sofield, R.M. | Chemical contaminant levels in edible seaweeds of the Salish Sea and implications for their consumption | 2022 | Plos one |
| Hardell, S.; Tilander, H.; Welfinger-Smith, G.; Burger, J.; Carpenter, D.O. | Levels of polychlorinated biphenyls (PCBs) and three organochlorine pesticides in fish from the Aleutian Islands of Alaska | 2010 | Plos one |
| Hlimi, T; Skinner, K; Hanning, RM; Martin, ID; Tsuji, LJS | Traditional food consumption behaviour and concern with environmental contaminants among Cree schoolchildren of the Mushkegowuk territory | 2012 | International journal of circumpolar health |
| Hu, X.F.; Laird, B.D.; Chan, H.M. | Mercury diminishes the cardiovascular protective effect of omega-3 polyunsaturated fatty acids in the modern diet of Inuit in Canada | 2017 | Environ. Res. |
| Irvine, G.; Doyle, J.R.; White, P.A.; Blais, J.M. | Soil ingestion rate determination in a rural population of Alberta, Canada practicing a wilderness lifestyle | 2014 | Sci. Total environ. |
| Jacques, A.D.; Furutani de Oliveira, M.A.; da Silva, M.C.; Hofer, C.B.; Basta, P.C. | Clinical, Laboratory and Neurodevelopmental Findings in Children from the Yanomami-Ninam Population Chronically Exposed to Methylmercury | 2024 | Toxics |
| Ji, XW; Abakumov, E; Polyakov, V | Assessments of pollution status and human health risk of heavy metals in permafrost-affected soils and lichens: A case-study in Yamal Peninsula, Russia Arctic | 2019 | Human and ecological risk assessment |
| Johnson-Down, L.; Labonte, M.E.; Martin, I.D.; Tsuji, L.J.S.; Nieboer, E.; Dewailly, E.; Egeland, G.; Lucas, M. | Quality of diet is associated with insulin resistance in the Cree (Eeyouch) indigenous population of northern Québec | 2015 | Nutr. Metab. Cardiovasc. Dis. |
| Johnson, LL; Ylitalo, GM; Myers, MS; Anulacion, BF; Buzitis, J; Collier, TK | Aluminum smelter-derived polycyclic aromatic hydrocarbons and flatfish health in the Kitimat marine ecosystem, British Columbia, Canada | 2015 | Science of the total environment |
| Jordan-Ward, R; von Hippel, FA; Zheng, GM; Salamova, A; Dillon, D; Gologergen, J; Immingan, T; Dominguez, E; Miller, P; Carpenter, D; Postlethwait, JH; Byrne, S; Buck, CL | Elevated mercury and PCB concentrations in Dolly Varden (Salvelinus malma) collected near a formerly used defense site on Sivuqaq, Alaska | 2022 | Science of the total environment |
| Jordan-Ward, R.; von Hippel, F.A.; Sancho Santos, M.E.; Wilson, C.A.; Rodriguez Maldonado, Z.; Dillon, D.; Titus, T.; Gardell, A.; Salamova, A.; Postlethwait, J.H.; Contreras, E.; Capozzi, S.L.; Panuwet, P.; Parrocha, C.; Bremiller, R.; Guiguen, Y.; Gologergen, J.; Immingan, T.; Miller, P.; Carpenter, D.; Buck, C.L. | Transcriptomic and developmental effects of persistent organic pollutants in sentinel fishes collected near an arctic formerly used defense site | 2024 | Environ. Pollut. |
| Jordan-Ward, R.; von Hippel, F.A.; Zheng, G.; Salamova, A.; Dillon, D.; Gologergen, J.; Immingan, T.; Dominguez, E.; Miller, P.; Carpenter, D.; Postlethwait, J.H.; Byrne, S.; Buck, C.L. | Elevated mercury and PCB concentrations in Dolly Varden (Salvelinus malma) collected near a formerly used defense site on Sivuqaq, Alaska | 2022 | Sci. Total environ. |
| Juric, A.K.; Batal, M.; David, W.; Sharp, D.; Schwartz, H.; Ing, A.; Fediuk, K.; Black, A.; Tikhonov, C.; Chan, H.M.; Chan, L. | Risk assessment of dietary lead exposure among First Nations people living on-reserve in Ontario, Canada using a total diet study and a probabilistic approach | 2018 | J. Hazard. Mater. |
| Juric, A.K.; Batal, M.; David, W.; Sharp, D.; Schwartz, H.; Ing, A.; Fediuk, K.; Black, A.; Tikhonov, C.; Chan, L.H.M. | A total diet study and probabilistic assessment risk assessment of dietary mercury exposure among First Nations living on-reserve in Ontario, Canada | 2017 | Environ. Res. |
| Kempton, J.W.; Périssé, A.R.S.; Hofer, C.B.; de Vasconcellos, A.C.S.; de Sousa Viana, P.V.; de Oliveira Lima, M.; de Jesus, I.M.; de Souza Hacon, S.; Basta, P.C. | An assessment of health outcomes and methylmercury exposure in munduruku indigenous women of childbearing age and their children under 2 years old | 2021 | Int. J. Environ. Res. Public health |
| Kenny, T.-A.; Hu, X.F.; Jamieson, J.A.; Kuhnlein, H.V.; Wesche, S.D.; Chan, H.M. | Potential impact of restricted caribou (Rangifer tarandus) consumption on anemia prevalence among Inuit adults in northern Canada | 2019 | Bmc nutr. |
| Krarup Hansen, K.; Turi, I.; Sundset, M.A.; Mathiesen, S.D. | Bridging traditional and scientific knowledge on reindeer meat smoking - a pilot study | 2022 | Int. J. Circumpolar health |
| Kwaansa-Ansah, E.E.; Basu, N.; Nriagu, J.O. | Environmental and occupational exposures to mercury among indigenous people in Dunkwa-On-Offin, a small scale gold mining area in the south-west of Ghana | 2010 | Bull. Environ. Contam. Toxicol. |
| Laffont, L.; Sonke, J.E.; Maurice, L.; Monrroy, S.L.; Chincheros, J.; Amouroux, D.; Behra, P. | Hg speciation and stable isotope signatures in human hair as a tracer for dietary and occupational exposure to mercury | 2011 | Environ. Sci. Technol. |
| Laird, B.D.; Chan, H.M. | Bioaccessibility of metals in fish, shellfish, wild game, and seaweed harvested in British Columbia, Canada | 2013 | Food chem. Toxicol. |
| Laird, BD; Goncharov, AB; Egeland, GM; Chan, HM | Dietary Advice on Inuit Traditional Food Use Needs to Balance Benefits and Risks of Mercury, Selenium, and n3 Fatty Acids | 2013 | Journal of nutrition |
| Laird, M.J.; Henao, J.J.A.; Reyes, E.S.; Stark, K.D.; Low, G.; Swanson, H.K.; Laird, B.D. | Mercury and omega-3 fatty acid profiles in freshwater fish of the Dehcho Region, Northwest Territories: Informing risk benefit assessments | 2018 | Sci. Total environ. |
| Lavers, J.L.; Bond, A.L. | Contaminants in indigenous harvests of apex predators: The Tasmanian Short-tailed Shearwater as a case study | 2013 | Ecotoxicol. Environ. Saf. |
| Liberda, E.N.; Tsuji, L.J.S.; Martin, I.D.; Ayotte, P.; Robinson, E.; Dewailly, E.; Nieboer, E. | Source identification of human exposure to lead in nine Cree Nations from Quebec, Canada (Eeyou Istchee territory) | 2018 | Environ. Res. |
| Liberda, E.N.; Tsuji, L.J.S.; Martin, I.D.; Cote, S.; Ayotte, P.; Dewailly, E.; Nieboer, E. | Plasma concentrations of persistent organic pollutants in the Cree of northern Quebec, Canada: Results from the multi-community environment-and-health study | 2014 | Sci. Total environ. |
| Liberda, E.N.; Wainman, B.C.; LeBlanc, A.; Dumas, P.; Martin, I.; Tsuji, L.J.S. | Dietary exposure of PBDEs resulting from a subsistence diet in three First Nation communities in the James Bay Region of Canada | 2011 | Environ. Int. |
| Liddell, J.L.; Kington, S.G.; Mckinley, C.E. | “We Live in a Very Toxic World”: Changing Environmental Landscapes and Indigenous Food Sovereignty | 2022 | Stud soc. Justice |
| Little, M.; Achouba, A.; Dumas, P.; Ouellet, N.; Ayotte, P.; Lemire, M. | Determinants of selenoneine concentration in red blood cells of Inuit from Nunavik (Northern Québec, Canada) | 2019 | Environ. Int. |
| Lucier, K.J.; Dickson-Anderson, S.E.; Skead, D.; Skead, K.; Kosmas, E.; Schuster-Wallace, C.J. | ‘That water out there is no damn good for anybody’: Experiences with declining water quality in a First Nation community | 2023 | Can water resour j |
| Makarov, D.A.; Komarov, A.A.; Ovcharenko, V.V.; Nebera, E.A.; Kozhushkevich, A.I.; Kalantaenko, A.M.; Afanasieva, E.L.; Demidova, S.V. | Dioxin and heavy metals contamination of reindeer offal from Russian far north regions | 2018 | Sel'skokhozyaistvennaya biol. |
| Martinez-Levasseur, L.M.; Simard, M.; Furgal, C.M.; Burness, G.; Bertrand, P.; Suppa, S.; Avard, E.; Lemire, M. | Towards a better understanding of the benefits and risks of country food consumption using the case of walruses in Nunavik (Northern Quebec, Canada) | 2020 | Sci. Total environ. |
| Marushka, L; Batal, M; Sadik, T; Schwartz, H; Ing, A; Fediuk, K; Tikhonov, C; Chan, HM | Seafood consumption patterns, their nutritional benefits and associated sociodemographic and lifestyle factors among First Nations in British Columbia, Canada | 2018 | Public health nutrition |
| Marushka, L; Hu, XF; Kenny, TA; Batal, M; Fediuk, K; Sadik, T; Golden, CD; Cheung, WWL; Salomon, AK; Chan, HM | Potential impacts of reduced seafood consumption on myocardial infarction among coastal First Nations in British Columbia, Canada | 2024 | Facets |
| Marushka, L.; Batal, M.; David, W.; Schwartz, H.; Ing, A.; Fediuk, K.; Sharp, D.; Black, A.; Tikhonov, C.; Chan, H.M. | Association between fish consumption, dietary omega-3 fatty acids and persistent organic pollutants intake, and type 2 diabetes in 18 First Nations in Ontario, Canada | 2017 | Environ. Res. |
| Marushka, L.; Hu, X.; Batal, M.; Sadik, T.; Schwartz, H.; Ing, A.; Fediuk, K.; Tikhonov, C.; Chan, H.M. | The relationship between persistent organic pollutants exposure and type 2 diabetes among first nations in Ontario and Manitoba, Canada: A difference in difference analysis | 2018 | Int. J. Environ. Res. Public health |
| Marushka, L.; Hu, X.; Batal, M.; Tikhonov, C.; Sadik, T.; Schwartz, H.; Ing, A.; Fediuk, K.; Chan, H.M. | The relationship between dietary exposure to persistent organic pollutants from fish consumption and type 2 diabetes among First Nations in Canada | 2021 | Can j public health |
| Matwee, L.; Pietrock, M. | Parasites and Metals in Walleye (Sander vitreus) and Northern Pike (Esox lucius) from Boreal Montreal Lake (Saskatchewan, Canada): Assessment of Human Health Risks | 2019 | Bull. Environ. Contam. Toxicol. |
| Maurice, L.; Barraza, F.; Blondet, I.; Ho-A-Chuck, M.; Tablon, J.; Brousse, P.; Demar, M.; Schreck, E. | Childhood lead exposure of Amerindian communities in French Guiana: an isotopic approach to tracing sources | 2021 | Environ. Geochem. Health |
| Mayor, P.; Soliño, L.; Cartró-Sabaté, M.; Orta-Martínez, M. | Impact of hydrocarbon extraction on heavy metal concentrations in lowland paca (Cuniculus paca) from the Peruvian Amazon | 2024 | Sci. Total environ. |
| McAuley, C.; Dersch, A.; Mouille-Malbeuf, S.; Koppe, B.; Sowan, D. | Cadmium Tissue Concentrations in Kidney, Liver and Muscle in Moose (Alces alces) From First Nations Communities in Northern Alberta | 2018 | Front. Sustain. food syst. |
| Medehouenou, T.C.M.; Larochelle, C.; Dumas, P.; Dewailly, É.; Ayotte, P. | Determinants of AhR-mediated transcriptional activity induced by plasma extracts from Nunavik Inuit adults | 2010 | Chemosphere |
| Mergler, D.; Philibert, A.; Fillion, M.; Da Silva, J. | The Contribution across Three Generations of Mercury Exposure to Attempted Suicide among Children and Youth in Grassy Narrows First Nation, Canada: An Intergenerational Analysis | 2023 | Environ. Health perspect. |
| Minick, D.J.; Paulik, L.B.; Smith, B.W.; Scott, R.P.; Kile, M.L.; Rohlman, D.; Anderson, K.A. | A passive sampling model to predict PAHs in butter clams (Saxidomus giganteus), a traditional food source for Native American tribes of the Salish Sea Region | 2019 | Mar. Pollut. Bull. |
| Mnisi, R.L.; Ndibewu, P.P.; Mafu, L.D.; Bwembya, G.C. | Bioaccessibility and risk assessment of essential and non-essential elements in vegetables commonly consumed in Swaziland | 2017 | Ecotoxicol. Environ. Saf. |
| Moriarity, R.J.; Ahmed, F.; Karagatzides, J.D.; Solomon, A.; Tsuji, L.J.S.; Liberda, E.N. | A cumulative human health risk assessment of the traditional diet of Indigenous youth in Western James Bay, Canada | 2024 | Hum. Ecol. Risk assess. |
| Moriarity, R.J.; Liberda, E.N.; Tsuji, L.J.S. | Subsistence fishing in the Eeyou Istchee (James Bay, Quebec, Canada): A regional investigation of fish consumption as a route of exposure to methylmercury | 2020 | Chemosphere |
| Moriarity, R.J.; Liberda, E.N.; Tsuji, L.J.S. | Using a geographic information system to assess local scale methylmercury exposure from fish in nine communities of the Eeyou Istchee territory (James Bay, Quebec, Canada) | 2020 | Environ. Res. |
| Moriarity, R.J.; Tsuji, L.J.S.; Liberda, E.N. | A probabilistic hazard and risk assessment of exposure to metals and organohalogens associated with a traditional diet in the Indigenous communities of Eeyou Istchee (northern Quebec, Canada) | 2023 | Environ. Sci. Pollut. Res. |
| Moriarity, R.J.; Zuk, A.M.; Liberda, E.N.; Tsuji, L.J.S. | Health measures of Eeyouch (Cree) who are eligible to participate in the on-the-land Income Security Program in Eeyou Istchee (northern Quebec, Canada) | 2021 | Bmc public health |
| Moriarity, RJ; Wilton, MJ; Liberda, EN; Tsuji, LJS; Peltier, RE | Wood smoke black carbon from Indigenous traditional cultural activities in a subarctic Cree community | 2020 | International journal of circumpolar health |
| Newman, J.; Behforooz, B.; Khuzwayo, A.G.; Gallo, M.V.; Schell, L.M. | PCBs and ADHD in Mohawk adolescents | 2014 | Neurotoxicol. Teratol. |
| Nieboer, E.; Martin, I.D.; Liberda, E.N.; Dewailly, E.; Robinson, E.; Tsuji, L.J.S. | Body burdens, sources and interrelations of selected toxic and essential elements among the nine Cree First Nations of: Eeyou Istchee, James Bay region of northern Quebec, Canada | 2017 | Environ. Sci. Process. Impacts |
| O'Callaghan-Gordo, C.; Rosales, J.; Lizárraga, P.; Barclay, F.; Okamoto, T.; Papoulias, D.M.; Espinosa, A.; Orta-Martinez, M.; Kogevinas, M.; Astete, J. | Blood lead levels in indigenous peoples living close to oil extraction areas in the Peruvian Amazon | 2021 | Environ. Int. |
| Olivero-Verbel, J.; Carranza-Lopez, L.; Caballero-Gallardo, K.; Ripoll-Arboleda, A.; Muñoz-Sosa, D. | Human exposure and risk assessment associated with mercury pollution in the Caqueta River, Colombian Amazon | 2016 | Environ. Sci. Pollut. Res. |
| Orta-Martínez, M.; Rosell-Melé, A.; Cartró-Sabaté, M.; O'Callaghan-Gordo, C.; Moraleda-Cibrián, N.; Mayor, P. | First evidences of Amazonian wildlife feeding on petroleum-contaminated soils: A new exposure route to petrogenic compounds? | 2018 | Environ. Res. |
| Packull-McCormick, S.; Cowan, A.; Stark, K.D.; Low, M.; Gamberg, M.; Swanson, H.; Laird, B. | Mercury bioaccessiblity in freshwater fish species from northern Canada | 2023 | Sci. Total environ. |
| Padhan, B.; Biswas, M.; Dhal, N.K.; Panda, D. | Evaluation of mineral bioavailability and heavy metal content in indigenous food plant wild yams (Dioscorea spp.) from Koraput, India | 2018 | J food sci technol |
| Panduro, G.; Rengifo, G.C.; Barreto, J.L.; Arbaiza-Peña, Á.K.; Iannacone, J.; Alvariño, L.; Crnobrna, B. | Bioaccumulation of mercury in fish and risk of ingestion in an indigenous community in the Peruvian Amazonia | 2020 | Rev. Investi. Vet. Peru |
| Pang, Y.; Peng, R.D.; Jones, M.R.; Francesconi, K.A.; Goessler, W.; Howard, B.V.; Umans, J.G.; Best, L.G.; Guallar, E.; Post, W.S.; Kaufman, J.D.; Vaidya, D.; Navas-Acien, A. | Metal mixtures in urban and rural populations in the US: The Multi-Ethnic Study of Atherosclerosis and the Strong Heart Study | 2016 | Environ. Res. |
| Paunescu, A.-C.; Ayotte, P.; Dewailly, E.; Dodin, S. | Dioxin-like compounds are not associated with bone strength measured by ultrasonography in Inuit women from Nunavik (Canada): Results of a cross-sectional study | 2013 | Int. J. Circumpolar health |
| Paunescu, A.-C.; Dewailly, E.; Dodin, S.; Nieboer, E.; Ayotte, P. | Dioxin-like compounds and bone quality in Cree women of Eastern James Bay (Canada): A cross-sectional study | 2013 | Environ. Health global access sci. Sour. |
| Peplow, D.; Augustine, S. | Neurological abnormalities in a mercury exposed population among indigenous Wayana in Southeast Suriname | 2014 | Environ. Sci. Process. Impacts |
| Perini, J.A.; Silva, M.C.; de Vasconcellos, A.C.S.; Viana, P.V.S.; Lima, M.O.; Jesus, I.M.; Kempton, J.W.; Oliveira, R.A.A.; Hacon, S.S.; Basta, P.C. | Genetic polymorphism of delta aminolevulinic acid dehydratase (Alad) gene and symptoms of chronic mercury exposure in munduruku indigenous children within the brazilian amazon | 2021 | Int. J. Environ. Res. Public health |
| Philibert, A.; Fillion, M.; Da Silva, J.; Lena, T.S.; Mergler, D. | Past mercury exposure and current symptoms of nervous system dysfunction in adults of a First Nation community (Canada) | 2022 | Environ. Health global access sci. Sour. |
| Philibert, A.; Fillion, M.; Mergler, D. | Mercury exposure and premature mortality in the Grassy Narrows First Nation community: a retrospective longitudinal study | 2020 | Lancet planet. Health |
| Phillips, N.R.; Stewart, M.; Olsen, G.; Hickey, C.W. | Human health risks of geothermally derived metals and other contaminants in wild-caught food | 2014 | J. Toxicol. Environ. Health part a curr. Iss. |
| Pontual, M.D.M.; Ayotte, P.; Little, M.; Furgal, C.; Boyd, A.D.; Muckle, G.; Avard, E.; Ricard, S.; Gauthier, M.-J.; Sidi, E.A.-L.; Lemire, M. | Seasonal variations in exposure to methylmercury and its dietary sources among pregnant Inuit women in Nunavik, Canada | 2021 | Sci. Total environ. |
| Ratelle, M.; Khoury, C.; Adlard, B.; Laird, B. | Polycyclic aromatic hydrocarbons (PAHs) levels in urine samples collected in a subarctic region of the Northwest Territories, Canada. | 2020 | Environ. Res. |
| Ratelle, M.; Laird, M.; Majowicz, S.; Skinner, K.; Swanson, H.; Laird, B. | Design of a human biomonitoring community-based project in the Northwest Territories Mackenzie Valley, Canada, to investigate the links between nutrition, contaminants and country foods | 2018 | Int. J. Circumpolar health |
| Ratelle, M.; Li, X.; Laird, B.D. | Cadmium exposure in First Nations communities of the Northwest Territories, Canada: smoking is a greater contributor than consumption of cadmium-accumulating organ meats | 2018 | Environ. Sci. Process. Impacts |
| Ratelle, M.; Skinner, K.; Laird, M.J.; Majowicz, S.; Brandow, D.; Packull-McCormick, S.; Bouchard, M.; Dieme, D.; Stark, K.D.; Henao, J.J.A.; Hanning, R.; Laird, B.D. | Implementation of human biomonitoring in the Dehcho region of the Northwest Territories, Canada (2016-2017) | 2018 | Arch. Public health |
| Reiner, J.L.; Becker, P.R.; Gribble, M.O.; Lynch, J.M.; Moors, A.J.; Ness, J.; Peterson, D.; Pugh, R.S.; Ragland, T.; Rimmer, C.; Rhoderick, J.; Schantz, M.M.; Trevillian, J.; Kucklick, J.R. | Organohalogen Contaminants and Vitamins in Northern fur Seals (Callorhinus ursinus) Collected during Subsistence Hunts in Alaska | 2016 | Arch. Environ. Contam. Toxicol. |
| Reyes, E.S.; Liberda, E.N.; Tsuji, L.J.S. | Human exposure to soil contaminants in subarctic Ontario, Canada | 2015 | Int. J. Circumpolar health |
| Ripley, S.; Robinson, E.; Johnson-Down, L.; Andermann, A.; Ayotte, P.; Lucas, M.; Nieboer, E. | Blood and hair mercury concentrations among Cree First Nations of Eeyou Istchee (Quebec, Canada): time trends, prenatal exposure and links to local fish consumption | 2018 | Int. J. Circumpolar health |
| Rivera, S.J.; Pacheco, L.F.; Achá, D.; Molina, C.I.; Miranda-Chumacero, G. | Low total mercury in Caiman yacare (Alligatoridae) as compared to carnivorous, and non-carnivorous fish consumed by Amazonian indigenous communities | 2016 | Environ. Pollut. |
| Rosell-Melé, A.; Moraleda-Cibrián, N.; Cartró-Sabaté, M.; Colomer-Ventura, F.; Mayor, P.; Orta-Martínez, M. | Oil pollution in soils and sediments from the Northern Peruvian Amazon | 2018 | Sci. Total environ. |
| Russell, S.; Sullivan, C.A.; Reichelt-Brushett, A.J. | Aboriginal consumption of estuarine food resources and potential implications for health through trace metal exposure; A study in Gumbaynggirr Country, Australia | 2015 | Plos one |
| Samuel-Nakamura, C.; Robbins, W.A.; Hodge, F.S. | Uranium and associated heavy metals in Ovis aries in a mining impacted area in northwestern New Mexico | 2017 | Int. J. Environ. Res. Public health |
| Sarkar, A.; Wilton, D.H.C.; Fitzgerald, E.; Sharma, A.; Sathya, A.J. | Environmental impact assessment of uranium exploration and development on indigenous land in Labrador (Canada): a community-driven initiative | 2019 | Environ. Geochem. Health |
| Scammell, M.K.; Sennett, C.; Laws, R.L.; Rubin, R.L.; Brooks, D.R.; Amador, J.J.; López-Pilarte, D.; Ramirez-Rubio, O.; Friedman, D.J.; McClean, M.D.; Lewis, J.; Erdei, E.; Begay, D.; Cajero, M.; Chavez, C.; Hoover, J.; Laselute, C.J.; Mackenzie, D.; O’donald, E.; Pacheco, B.; Smith, B.; Anderson, Q.; Begay, M.-G.; Begay, N.; Harold, V.; Muskett, O.; Rondon, A.; Thompson, R.; Tsinnijinnie, D.; Tsosie, R.; Watson, J.; Atene, L.; Barton, L.; Begay, F.; Begay, P.; Benally, D.; Chee, B.; Clawson, B.; Crank, L.; Francisco, M.; Kear, L.; Knoki-Wilson, U.; Reese, D.; Rogers, J.; Sanders, A.; Sam, D.; Samuel, M.; Swindal, C.; Tapaha, M.; Lasiloo, L.; Nez, T.; Phillips, C.; Ramone, S.; Shuey, C.; Welch, M.; Navajo Birth Cohort Study Team | Urinary metals concentrations and biomarkers of autoimmunity among navajo and nicaraguan men | 2020 | Int. J. Environ. Res. Public health |
| Schartup, A.T.; Balcom, P.H.; Soerensen, A.L.; Gosnell, K.J.; Calder, R.S.D.; Mason, R.P.; Sunderland, E.M.; St. Louis, V.L. | Freshwater discharges drive high levels of methylmercury in Arctic marine biota | 2015 | Proc. Natl. Acad. Sci. U. S. A. |
| Schuster, R.C.; Gamberg, M.; Dickson, C.; Chan, H.M. | Assessing risk of mercury exposure and nutritional benefits of consumption of caribou (Rangifer tarandus) in the Vuntut Gwitchin First Nation community of Old Crow, Yukon, Canada | 2011 | Environ. Res. |
| Seabert, T.A.; Pal, S.; Pinet, B.M.; Haman, F.; Robidoux, M.A.; Imbeault, P.; Krümmel, E.M.; Kimpe, L.E.; Blais, J.M. | Elevated contaminants contrasted with potential benefits of ω-3 fatty acids in wild food consumers of two remote first nations communities in Northern Ontario, Canada | 2014 | Plos one |
| Silva, M.C.D.; Basta, P.C.; Hofer, C.B.; Oliveira, M.A.F.D.; Kempton, J.W.; Oliveira, R.A.A.D.; Vasconcellos, A.C.S.D.; Perini, J.A. | The GSTP1 rs1695 Polymorphism Is Associated with Mercury Levels and Neurodevelopmental Delay in Indigenous Munduruku Children from the Brazilian Amazon | 2024 | Toxics |
| Simon, S.L.; Bouville, A.; Beck, H.L.; Melo, D.R. | Estimated radiation doses received by New Mexico residents from the 1945 Trinity nuclear test | 2020 | Health phys. |
| Simpson, A.K.; Drysdale, M.; Gamberg, M.; Froese, K.; Brammer, J.; Dumas, P.; Ratelle, M.; Skinner, K.; Laird, B.D. | Human biomonitoring of dioxins, furans, and non-ortho dioxin-like polychlorinated biphenyls (PCBs) in blood plasma from Old Crow, Yukon, Canada (2019) | 2024 | Sci. Total environ. |
| Skalny, AV; Mona, W; Kao, R; Skalnaya, MG; Huang, PT; Wu, CC; Ajsuvakova, OP; Skalnaya, OA; Tinkov, AA | Hair Trace Element Levels in Han and Indigenous Hualien Inhabitants in Taiwan | 2019 | Biological trace element research |
| Smith, C.D.; Payne, S.E.; Morace, J.L.; Nilsen, E.B. | Organohalogenated contaminants in multiple life stages of the Pacific lamprey (Entosphenus tridentatus), Oregon, USA | 2023 | Environ. Pollut. |
| Sobolev, N; Ellingsen, DG; Belova, N; Aksenov, A; Sorokina, T; Trofimova, A; Varakina, Y; Kotsur, D; Grjibovski, AM; Chashchin, V; Bogolitsyn, K; Thomassen, Y | Essential and non-essential elements in biological samples of inhabitants residing in Nenets Autonomous Okrug of the Russian Arctic | 2021 | Environment international |
| Somnath Bhowmik; Datta, B. K.; Saha, A. K. | Determination of mineral content and heavy metal content of some traditionally important aquatic plants of Tripura, India using atomic absorption spectroscopy. | 2012 | International journal of agricultural technology |
| Sonne, C.; Desforges, J.-P.; Gustavson, K.; Bossi, R.; Bonefeld-Jørgensen, E.C.; Long, M.; Rigét, F.F.; Dietz, R. | Assessment of exposure to perfluorinated industrial substances and risk of immune suppression in Greenland and its global context: a mixed-methods study | 2023 | Lancet planet. Health |
| Sorokina, T.; Sobolev, N.; Belova, N.; Aksenov, A.; Kotsur, D.; Trofimova, A.; Varakina, Y.; Grjibovski, A.M.; Chashchin, V.; Korobitsyna, R.; Thomassen, Y. | Diet and Blood Concentrations of Essential and Non-Essential Elements among Rural Residents in Arctic Russia | 2022 | Nutrients |
| Souza-Araujo, J.; Giarrizzo, T.; Lima, M.O.; Souza, M.B. | Mercury and methyl mercury in fishes from Bacajá River (Brazilian Amazon): evidence for bioaccumulation and biomagnification | 2016 | J. Fish biol. |
| St. Pierre, K.A.; St. Louis, V.L.; Kirk, J.L.; Lehnherr, I.; Wang, S.; La Farge, C. | Importance of open marine waters to the enrichment of total mercury and monomethylmercury in lichens in the canadian high arctic | 2015 | Environ. Sci. Technol. |
| Stachiw, S.; Bicalho, B.; Grant-Weaver, I.; Noernberg, T.; Shotyk, W. | Trace elements in berries collected near upgraders and open pit mines in the Athabasca Bituminous Sands Region (ABSR): Distinguishing atmospheric dust deposition from plant uptake | 2019 | Sci. Total environ. |
| Stewart, M.; Phillips, N.R.; Olsen, G.; Hickey, C.W.; Tipa, G. | Organochlorines and heavy metals in wild caught food as a potential human health risk to the indigenous MĀori population of South Canterbury, New Zealand | 2011 | Sci. Total environ. |
| Stroink, M. L.; Nelson, C. H. | Understanding traditional food behaviour and food security in rural First Nation communities: implications for food policy. | 2012 | Journal of rural and community development |
| Suami, R.B.; Al Salah, D.M.M.; Kabala, C.D.; Otamonga, J.-P.; Mulaji, C.K.; Mpiana, P.T.; Poté, J.W. | Assessment of metal concentrations in oysters and shrimp from Atlantic Coast of the Democratic Republic of the Congo | 2019 | Heliyon |
| Tam, B.; Tsuji, L.J.S.; Martin, I.D.; Liberda, E.N.; Ayotte, P.; Coté, S.; Dewailly, E.; Nieboer, E. | Iodine status of Eeyou Istchee community members of northern Quebec, Canada, and potential sources | 2015 | Environ. Sci. Process. Impacts |
| Tanamal, C; Blais, JM; Yumvihoze, E; Chan, HM | Health risk assessment of inorganic arsenic exposure through fish consumption in Yellowknife, Northwest Territories, Canada | 2021 | Human and ecological risk assessment |
| Thompson, K.-L.; Picard, C.R.; Chan, H.M. | Polycyclic aromatic hydrocarbons (PAHs) in traditionally harvested bivalves in northern British Columbia, Canada | 2017 | Mar. Pollut. Bull. |
| Tian, WJ; Egeland, GM; Sobol, I; Chan, HM | Mercury hair concentrations and dietary exposure among Inuit preschool children in Nunavut, Canada | 2011 | Environment international |
| Tikhonov, C; Schwartz, H; Marushka, L; Chan, HM; Batal, M; Sadik, T; Ing, A; Fediuk, K | Regionally representative hair mercury levels in Canadian First Nations adults living on reserves | 2021 | Canadian journal of public health-revue canadienne de sante publique |
| Trukhin, AM; Simokon, MV | Mercury in organs of Pacific walruses (Odobenus rosmarus divergens) from the Bering Sea | 2018 | Environmental science and pollution research |
| Unguryanu, T.N.; Lyzhina, A.V.; Mitrokhin, O.V.; Polibin, R.V. | Human health risk assessment of heavy metals from meat and offal of reindeer and cow in the Far North of European Russia | 2023 | Scand. J. Public health |
| Valdelamar-Villegas, J.; Olivero-Verbel, J. | High Mercury Levels in the Indigenous Population of the Yaigojé Apaporis National Natural Park, Colombian Amazon | 2020 | Biol. Trace elem. Res. |
| Valera, B.; Dewailly, E.; Poirier, P. | Impact of mercury exposure on blood pressure and cardiac autonomic activity among Cree adults (James Bay, Quebec, Canada) | 2011 | Environ. Res. |
| Valera, B.; Dewailly, T.; Poirier, P. | Association between methylmercury and cardiovascular risk factors in a native population of Quebec (Canada): A retrospective evaluation | 2013 | Environ. Res. |
| Valera, B.; Jørgensen, M.E.; Jeppesen, C.; Bjerregaard, P. | Exposure to persistent organic pollutants and risk of hypertension among Inuit from Greenland | 2013 | Environ. Res. |
| Van Horne, Y.O.; Chief, K.; Charley, P.H.; Begay, M.-G.; Lothrop, N.; Canales, R.A.; Beamer, P.I. | A Community-Based Health Risk Assessment Following the Gold King Mine Spill: Results from the Gold King Mine Spill Diné Exposure Project | 2024 | Expos. Health |
| Varakina, Y; Aksenov, A; Lakhmanov, D; Trofimova, A; Korobitsyna, R; Belova, N; Kotsur, D; Sorokina, T; Grjibovski, AM; Popova, L; Chashchin, V; Odland, JO; Thomassen, Y | Geographic and Ethnic Variations in Serum Concentrations of Legacy Persistent Organic Pollutants among Men in the Nenets Autonomous Okrug, Arctic Russia | 2022 | International journal of environmental research and public health |
| Varty, S.; Lehnherr, I.; St. Pierre, K.; Kirk, J.; Wisniewski, V. | Methylmercury Transport and Fate Shows Strong Seasonal and Spatial Variability along a High Arctic Freshwater Hydrologic Continuum | 2021 | Environ. Sci. Technol. |
| Vega, C.M.; Orellana, J.D.Y.; Oliveira, M.W.; Hacon, S.S.; Basta, P.C. | Human mercury exposure in yanomami indigenous villages from the Brazilian Amazon | 2018 | Int. J. Environ. Res. Public health |
| Velásquez Ramírez, M.G.; Vega Ruiz, C.M.; Gomringer, R.C.; Pillaca, M.; Thomas, E.; Stewart, P.M.; Gamarra Miranda, L.A.; Dañobeytia, F.R.; Guerrero Barrantes, J.A.; Gushiken, M.C.; Bardales, J.V.; Silman, M.; Fernandez, L.; Ascorra, C.; Torres, D.D.C. | Mercury in soils impacted by alluvial gold mining in the Peruvian Amazon | 2021 | J. Environ. Manage. |
| von Hippel, F.A.; Miller, P.K.; Carpenter, D.O.; Dillon, D.; Smayda, L.; Katsiadaki, I.; Titus, T.A.; Batzel, P.; Postlethwait, J.H.; Buck, C.L. | Endocrine disruption and differential gene expression in sentinel fish on St. Lawrence Island, Alaska: Health implications for indigenous residents | 2018 | Environ. Pollut. |
| Walker, V.K.; Das, P.; Li, P.; Lougheed, S.C.; Moniz, K.; Schott, S.; Qitsualik, J.; Koch, I. | Identification of arctic food fish species for anthropogenic contaminant testing using geography and genetics | 2020 | Foods |
| Watson, L.C.; Hurtado-Gonzales, J.L.; Chin, C.J.; Persaud, J. | Survey of methylmercury exposures and risk factors among Indigenous communities in Guyana, South America | 2020 | J. Health pollut. |
| Webb, J.; Coomes, O.T.; Ross, N.; Mergler, D. | Mercury concentrations in urine of amerindian populations near oil fields in the peruvian and ecuadorian amazon | 2016 | Environ. Res. |
| Weinhouse, C.; Gallis, J. A.; Ortiz, E.; Berky, A. J.; Morales, A. M.; Diringer, S. E.; Harrington, J.; Bullins, P.; Rogers, L.; Hare-Grogg, J.; Hsu-Kim, H.; Pan, W. K. | A population-based mercury exposure assessment near an artisanal and small-scale gold mining site in the Peruvian Amazon. | 2020 | Journal of exposure science and environmental epidemiology |
| Wyatt, L; Permar, SR; Ortiz, E; Berky, A; Woods, CW; Amouou, GF; Itell, H; Hsu-Kim, H; Pan, W | Mercury Exposure and Poor Nutritional Status Reduce Response to Six Expanded Program on Immunization Vaccines in Children: An Observational Cohort Study of Communities Affected by Gold Mining in the Peruvian Amazon | 2019 | International journal of environmental research and public health |
