# Supplementary material for: Community engagement in Indigenous food systems contamination studies: A systematic scoping review
Source: PLoS One. 2025 Nov 14;20(11):e0336439. doi: 10.1371/journal.pone.0336439 (PMC12617898; doi:10.1371/journal.pone.0336439)
Supplement: S2 Table — (DOCX) [file pone.0336439.s002.docx]

| **Study ID** | **Indigenous**  **Affiliation** | **Methods** | **Indigenous Population** | **Indigenous**  **Group** | **Country** | **Community**  **Engagement Activities** | **Community**  **use of**  **Study Results** |
| --- | --- | --- | --- | --- | --- | --- | --- |
| Achatz 2021 | No | Biomonitoring human samples; Quantitative observational study; Qualitative | Munduruku | Amazon Indigenous Peoples | Brazil | Research requested by Indigenous population; Community members participated conducting research activities | Study results are expected to inform an specific broader strategy, program, or intervention to control exposure, improve environmental quality, or restore food system |
| Achouba 2016 | No | Biomonitoring human samples; Secondary data analysis / Previously collected data | Inuit | Circumpolar | Canada | Not mentioned | Not mentioned |
| Adamou 2018 | No | Biomonitoring human samples; Quantitative observational study | Nunavimmiut | Circumpolar | Canada | Not mentioned | Not mentioned |
| Adamou 2020 | No | Biomonitoring human samples; Secondary data analysis / Previously collected data; Quantitative observational study | Nunavimmiut | Circumpolar | Canada | Activity to receive community feedback | Not mentioned |
| Adams 2019 | No | Biomonitoring food samples | Qawalangin Tribe | Native American | United States | Research requested by Indigenous population | Study results are expected to inform an specific broader strategy, program, or intervention to control exposure, improve environmental quality, or restore food system |
| Adhikari 2021 | No | Biomonitoring food samples | Bapedi | Bapedi | South Africa | Not mentioned | Not mentioned |
| Akbar 2021 | No | Biomonitoring human samples; Secondary data analysis / Previously collected data; Quantitative observational study | Cree First Nations | First Nations | Canada | Information session conducted; Activity to receive community feedback | Not mentioned |
| Aker 2022 | Yes | Biomonitoring human samples; Secondary data analysis / Previously collected data | Nunavimmiut | Circumpolar | Canada | Not mentioned | Not mentioned |
| Aker 2023 | Yes | Biomonitoring human samples; Secondary data analysis / Previously collected data; Quantitative observational study; Demographics questionnaire | Inuit | Circumpolar | Canada | Community members participated conducting research activities; Research methods were designed in collaboration with Indigenous Population members; Results discussion were conducted in collaboration with Indigenous Population members | Not mentioned |
| Aker 2023 | Yes | Biomonitoring human samples; Secondary data analysis / Previously collected data; Quantitative observational study; Demographics questionnaire | Inuit | Circumpolar | Canada | Community members participated conducting research activities; Research methods were designed in collaboration with Indigenous Population members; Results discussion were conducted in collaboration with Indigenous Population members | Not mentioned |
| AkwesasneTaskForceEnvironm 2016 | Yes | Biomonitoring human samples; Quantitative observational study | Mohawk Nation | Native American | United States | Community members participated conducting research activities | Not mentioned |
| Alcala-Orozco 2019 | No | Biomonitoring human samples; Quantitative observational study | Tikuna, Uitoto, Bora, Ocaina | Amazon Indigenous Peoples | Colombia | Information session conducted | Not mentioned |
| Anbleyth-Evans 2020 | No | Environmental monitoring; Qualitative | Mapuche-Huichille | Mapuche | Chile | Information session conducted; Activity to receive community feedback; Community members participated conducting research activities | Study results are expected to inform an specific broader strategy, program, or intervention to control exposure, improve environmental quality, or restore food system |
| Andrade-Rivas 2022 | Yes | Biomonitoring food samples; Environmental monitoring | Tsleil-Waututh Nation | First Nations | Canada | Research requested by Indigenous population; Community members participated conducting research activities; Research methods were designed in collaboration with Indigenous Population members; Results discussion were conducted in collaboration with Indigenous Population members | Study results are already part of a broader strategy, program, or intervention to control exposure, improve environmental quality, or restore food system |
| Anticona 2012 | No | Biomonitoring human samples; Quantitative observational study | Not specified | Amazon Indigenous Peoples | Peru | Information session conducted | Not mentioned |
| Arrifano 2018 | No | Biomonitoring human samples; Quantitative observational study | Not specified | Amazon Indigenous Peoples | Brazil | Not mentioned | Not mentioned |
| Baldwin 2018 | No | Environmental monitoring; Secondary data analysis / Previously collected data; Qualitative ; Indigenous knowledge and Western sciences were integrated to develop methods | First Nations and Metis | First Nations; Metis | Canada | Activity to receive community feedback; Community members participated conducting research activities | Not mentioned |
| Bank-Nielsen 2019 | No | Secondary data analysis / Previously collected data; Quantitative observational study | Inuit | Circumpolar | Greenland | Not mentioned | Not mentioned |
| Basta 2021 | No | Biomonitoring human samples; Biomonitoring food samples; Quantitative observational study | Munduruku (Sawré Muybu, Poxo Muybu, and Sawré Aboy) | Amazon Indigenous Peoples | Brazil | Activity to receive community feedback; Community members participated conducting research activities | Not mentioned |
| Benefice 2010 | No | Biomonitoring human samples; Quantitative observational study | Takanas and Esse Ejjas | Amazon Indigenous Peoples | Bolivia | Not mentioned | Not mentioned |
| Berky 2022 | No | Biomonitoring human samples; Secondary data analysis / Previously collected data; Quantitative observational study; Demographics questionnaire | Queros, Palotoa Teparo, Shintuya, Shipetiari, Diamante, Boca Manu, Puerto Azul, Boca Isiriwe, Masenawa, Puerto Luz, San Lorenzo, Tres Islas | Amazon Indigenous Peoples | Peru | Not mentioned | Not mentioned |
| Bhattacharya 2023 | No | Biomonitoring food samples | Rabhas, Sonowal Kacharis | Tribes of Assam | India | Not mentioned | Not mentioned |
| Binnington 2016 | No | Environmental monitoring; Secondary data analysis / Previously collected data; Other: Simulation and modelling | Inuit | Circumpolar; First Nations; Metis | Canada | Not mentioned | Not mentioned |
| Binnington 2016 | No | Biomonitoring human samples; Environmental monitoring; Secondary data analysis / Previously collected data; Quantitative observational study; Other: Simulation and modelling | Inuit, Dene, and Métis | Circumpolar; First Nations; Metis | Canada | Not mentioned | Not mentioned |
| Binnington 2017 | Yes | Biomonitoring food samples | Inuvialuit | Circumpolar | Canada | Community members participated conducting research activities | Not mentioned |
| Blanco 2020 | No | Qualitative ; Demographics questionnaire | Not specified | Not specified | Brazil | Not mentioned | Not mentioned |
| Bordeleau 2016 | No | Biomonitoring food samples; Quantitative observational study; Qualitative | Anishnaabeg | First Nations | Canada | Activity to receive community feedback; Research requested by Indigenous population; Community members participated conducting research activities; Research methods were designed in collaboration with Indigenous Population members | Study results are expected to inform an specific broader strategy, program, or intervention to control exposure, improve environmental quality, or restore food system |
| Boucher 2016 | No | Biomonitoring human samples; Quantitative observational study | Inuit | Circumpolar | Canada | Not mentioned | Not mentioned |
| Brunet 2020 | Yes | Biomonitoring food samples; Qualitative ; Indigenous knowledge and Western sciences were integrated to develop methods; Demographics questionnaire | Cold Lake First Nations | First Nations | Canada | Information session conducted; Activity to receive community feedback; Research requested by Indigenous population; Community members participated conducting research activities; Research methods were designed in collaboration with Indigenous Population members; Results discussion were conducted in collaboration with Indigenous Population members | Study results are already part of a broader strategy, program, or intervention to control exposure, improve environmental quality, or restore food system |
| Byrne 2017 | No | Biomonitoring human samples; Biomonitoring food samples; Environmental monitoring | Yupik | Circumpolar | United States | Not mentioned | Not mentioned |
| Byrne 2018 | No | Biomonitoring human samples; Quantitative observational study | Yupik | Circumpolar | United States | Not mentioned | Not mentioned |
| Byrne 2022 | Yes | Biomonitoring food samples | Sivuqaq Yupik | Circumpolar | United States | Community members participated conducting research activities; Research methods were designed in collaboration with Indigenous Population members; Results discussion were conducted in collaboration with Indigenous Population members | Not mentioned |
| Calder 2019 | No | Biomonitoring human samples; Secondary data analysis / Previously collected data; Quantitative observational study;Other: Simulation and modelling | Inuit | Circumpolar | Canada | Activity to receive community feedback; Community members participated conducting research activities | Study results are expected to inform an specific broader strategy, program, or intervention to control exposure, improve environmental quality, or restore food system |
| Caron-Beaudoin 2019 | No | Biomonitoring human samples; Secondary data analysis / Previously collected data; Qualitative | First Nation | First Nations | Canada | Information session conducted; Activity to receive community feedback | Not mentioned |
| Chaisson 2012 | No | Secondary data analysis / Previously collected data; Qualitative | Inupiaq (Selawik) | Circumpolar | United States | Information session conducted; Activity to receive community feedback; Research requested by Indigenous population; Community members participated conducting research activities | Study results are expected to inform an specific broader strategy, program, or intervention to control exposure, improve environmental quality, or restore food system |
| Champeau 2020 | No | Environmental monitoring | Māori | Maori | New Zealand | Not mentioned | Not mentioned |
| Charania 2014 | No | Biomonitoring human samples; Secondary data analysis / Previously collected data; Quantitative observational study | Cree First Nations | First Nations | Canada | Activity to receive community feedback; Community members participated conducting research activities | Study results are already part of a broader strategy, program, or intervention to control exposure, improve environmental quality, or restore food system |
| Charette 2021 | No | Secondary data analysis / Previously collected data; Other: Simulation and modelling | Inuit | Circumpolar | Canada | Not mentioned | Not mentioned |
| Chashchin 2020 | No | Biomonitoring human samples; Environmental monitoring; Secondary data analysis / Previously collected data; Quantitative observational study | Not specified | Circumpolar | Russia | Not mentioned | Not mentioned |
| Cooke 2022 | No | Biomonitoring food samples | First Nations British Columbia | First Nations | Canada | Community members participated conducting research activities | Not mentioned |
| Cordier 2020 | No | Biomonitoring human samples; Secondary data analysis / Previously collected data; Quantitative observational study | Inuit and Cree First Nations | Circumpolar; First Nations | Canada | Not mentioned | Not mentioned |
| Cott 2018 | Yes | Biomonitoring food samples; Qualitative ; Indigenous research methods | Aklavik, Fort Mcpherson, Inuvik, Tsiigehtchic | Circumpolar | Canada | Activity to receive community feedback; Community members participated conducting research activities; Research methods were designed in collaboration with Indigenous Population members | Study results are expected to inform an specific broader strategy, program, or intervention to control exposure, improve environmental quality, or restore food system |
| Curren 2014 | No | Biomonitoring human samples; Secondary data analysis / Previously collected data; Demographics questionnaire | Inuit | Circumpolar | Canada | Not mentioned | Not mentioned |
| Curren 2015 | No | Biomonitoring human samples; Quantitative observational study | Inuit, Dene, and Metis | Circumpolar; First Nations; Metis | Canada | Not mentioned | Not mentioned |
| daSilva 2023 | No | Biomonitoring food samples; Environmental monitoring | Kayapó | Amazon Indigenous Peoples | Brazil | Community members participated conducting research activities | Not mentioned |
| deBakker 2021 | No | Biomonitoring human samples; Secondary data analysis / Previously collected data; Other: Simulation and modelling | Yanomami | Amazon Indigenous Peoples | Brazil | Not mentioned | Not mentioned |
| Deere 2020 | Yes | Biomonitoring food samples; Environmental monitoring | Anishinaabeg | Native American | United States | Information session conducted; Activity to receive community feedback; Research requested by Indigenous population; Research methods were designed in collaboration with Indigenous Population members | Study results are expected to inform an specific broader strategy, program, or intervention to control exposure, improve environmental quality, or restore food system |
| Deere 2024 | Yes | Biomonitoring food samples | Grand Portage Band of Lake Superior Chippewa (Chippewa) | Native American | United States | Not mentioned | Not mentioned |
| Dellinger 2016 | Yes | Biomonitoring food samples; Secondary data analysis / Previously collected data; Indigenous knowledge and Western sciences were integrated to develop methods | Anishinaabe | Native American | United States | Research requested by Indigenous population; Community members participated conducting research activities; Research methods were designed in collaboration with Indigenous Population members | Study results are already part of a broader strategy, program, or intervention to control exposure, improve environmental quality, or restore food system |
| Dellinger 2018 | Yes | Biomonitoring food samples; Secondary data analysis / Previously collected data; Qualitative ; Indigenous knowledge and Western sciences were integrated to develop methods; Other: Simulation and modelling | Anishinaabe | Native American | United States | Activity to receive community feedback; Community members participated conducting research activities; Research methods were designed in collaboration with Indigenous Population members; Results discussion were conducted in collaboration with Indigenous Population members | Study results are expected to inform an specific broader strategy, program, or intervention to control exposure, improve environmental quality, or restore food system |
| Dellinger 2018 | Yes | Biomonitoring food samples; Secondary data analysis / Previously collected data; Indigenous knowledge and Western sciences were integrated to develop methods | Anishinaabe | Native American | United States | Activity to receive community feedback; Research requested by Indigenous population; Community members participated conducting research activities; Research methods were designed in collaboration with Indigenous Population members | Study results are already part of a broader strategy, program, or intervention to control exposure, improve environmental quality, or restore food system |
| Dellinger 2019 | Yes | Quantitative observational study; Qualitative ; Indigenous knowledge and Western sciences were integrated to develop methods | Anishinaabeg | Native American | United States | Activity to receive community feedback; Community members participated conducting research activities; Research methods were designed in collaboration with Indigenous Population members | Study results are already part of a broader strategy, program, or intervention to control exposure, improve environmental quality, or restore food system |
| Dellinger 2020 | Yes | Biomonitoring food samples; Secondary data analysis / Previously collected data; Quantitative observational study;Other: Simulation and modelling | Anishinaabe | Native American | United States | Information session conducted; Activity to receive community feedback; Community members participated conducting research activities; Research methods were designed in collaboration with Indigenous Population members | Study results are already part of a broader strategy, program, or intervention to control exposure, improve environmental quality, or restore food system |
| Dellinger 2022 | Yes | Biomonitoring food samples; Secondary data analysis / Previously collected data; Quantitative observational study; Indigenous knowledge and Western sciences were integrated to develop methods; Demographics questionnaire | Anishinaabe | Native American | United States | Activity to receive community feedback; Community members participated conducting research activities; Research methods were designed in collaboration with Indigenous Population members; Results discussion were conducted in collaboration with Indigenous Population members | Study results are already part of a broader strategy, program, or intervention to control exposure, improve environmental quality, or restore food system |
| DeLoma 2019 | No | Biomonitoring human samples; Environmental monitoring; Quantitative observational study | Uru and Aymara-Quechua | Andean Indigenous Peoples | Bolivia | Information session conducted | Not mentioned |
| deMatos 2018 | No | Biomonitoring food samples | Not specified | Amazon Indigenous Peoples | Brazil | Not mentioned | Not mentioned |
| deOliveira 2021 | No | Biomonitoring food samples | Tupari | Amazon Indigenous Peoples | Brazil | Not mentioned | Not mentioned |
| deVasconcellos 2021 | No | Biomonitoring food samples; Quantitative observational study | Munduruku | Amazon Indigenous Peoples | Brazil | Not mentioned | Not mentioned |
| Diringer 2015 | No | Biomonitoring food samples; Environmental monitoring | Not specified | Amazon Indigenous Peoples | Peru | Not mentioned | Not mentioned |
| Doyle 2012 | No | Qualitative ; Demographics questionnaire | Xeni Gwet'in First Nations | First Nations | Canada | Activity to receive community feedback; Community members participated conducting research activities | Not mentioned |
| Doyle 2012 | No | Biomonitoring human samples; Biomonitoring food samples; Environmental monitoring | Xeni Gwet'in First Nation | First Nations | Canada | Information session conducted; Activity to receive community feedback | Not mentioned |
| Drescher 2014 | No | Biomonitoring human samples; Quantitative observational study | Cree | First Nations | Canada | Not mentioned | Not mentioned |
| Driscoll 2012 | No | Qualitative | Lumbee Tribe | Native American | United States | Not mentioned | Study results are expected to inform an specific broader strategy, program, or intervention to control exposure, improve environmental quality, or restore food system |
| Drysdale 2021 | Yes | Biomonitoring human samples; Demographics questionnaire | Vuntut Gwitchin First Nation | First Nations | Canada | Information session conducted; Activity to receive community feedback; Research requested by Indigenous population; Community members participated conducting research activities; Results discussion were conducted in collaboration with Indigenous Population members | Study results are already part of a broader strategy, program, or intervention to control exposure, improve environmental quality, or restore food system |
| Drysdale 2023 | Yes | Biomonitoring human samples; Secondary data analysis / Previously collected data; Quantitative observational study; Demographics questionnaire | Gwich’in, Dene | First Nations | Canada | Information session conducted; Activity to receive community feedback; Research requested by Indigenous population; Community members participated conducting research activities; Results discussion were conducted in collaboration with Indigenous Population members | Study results are already part of a broader strategy, program, or intervention to control exposure, improve environmental quality, or restore food system |
| Drysdale 2024 | Yes | Biomonitoring human samples; Secondary data analysis / Previously collected data; Quantitative observational study; Demographics questionnaire | Gwitch'in | First Nations | Canada | Activity to receive community feedback; Research requested by Indigenous population; Community members participated conducting research activities; Research methods were designed in collaboration with Indigenous Population members; Results discussion were conducted in collaboration with Indigenous Population members | Study results are already part of a broader strategy, program, or intervention to control exposure, improve environmental quality, or restore food system |
| Dudarev 2010 | No | Biomonitoring human samples; Quantitative observational study | Chukchy and Inuit | Circumpolar | Russia | Not mentioned | Not mentioned |
| Dudarev 2019 | No | Secondary data analysis / Previously collected data; Quantitative observational study; Qualitative | Yupik, Chukchi, and Inupiaq | Circumpolar | Russia; United States | Not mentioned | Study results are expected to inform an specific broader strategy, program, or intervention to control exposure, improve environmental quality, or restore food system |
| El-DinBekhit 2011 | No | Biomonitoring food samples | Māori | Pacific Islander | New Zealand | Not mentioned | Not mentioned |
| Forsberg 2012 | Yes | Biomonitoring food samples; Environmental monitoring | Confederated Tribes of the Umatilla Indian Reservation | Native American | United States | Research requested by Indigenous population; Community members participated conducting research activities; Research methods were designed in collaboration with Indigenous Population members; Results discussion were conducted in collaboration with Indigenous Population members | Study results are already part of a broader strategy, program, or intervention to control exposure, improve environmental quality, or restore food system |
| Furgal 2023 | No | Quantitative observational study; Qualitative; Demographics questionnaire | Inuit | Circumpolar | Canada | Community members participated conducting research activities | Study results are expected to inform an specific broader strategy, program, or intervention to control exposure, improve environmental quality, or restore food system |
| Gadamus 2013 | No | Qualitative ; Indigenous knowledge and Western sciences were integrated to develop methods | Inupiat, Yup’ik and St. Lawrence Island Yupik residents | Circumpolar | United States | Information session conducted; Activity to receive community feedback; Community members participated conducting research activities | Study results are expected to inform an specific broader strategy, program, or intervention to control exposure, improve environmental quality, or restore food system |
| Gallo 2011 | Yes | Biomonitoring human samples; Quantitative observational study | Akwesasne Mohawk Nation | Native American | United States | Activity to receive community feedback; Research requested by Indigenous population; Community members participated conducting research activities | Study results are already part of a broader strategy, program, or intervention to control exposure, improve environmental quality, or restore food system |
| Gao 2020 | No | Secondary data analysis / Previously collected data; Quantitative observational study; Other: Simulation and modelling | Not specified | Circumpolar | Russia | Not mentioned | Not mentioned |
| Garcia-Barrios 2021 | No | Biomonitoring human samples; Demographics questionnaire | First Nations (Old Crow, Yukon and K’atl’odeeche, Deh Gah Gotie, Ka’a’gee Tu, Sambaa Ke, Jean Marie River First Nation, and West Point First Nation) | First Nations | Canada | Activity to receive community feedback; Community members participated conducting research activities; Research methods were designed in collaboration with Indigenous Population members | Study results are expected to inform an specific broader strategy, program, or intervention to control exposure, improve environmental quality, or restore food system |
| Garry 2018 | No | Biomonitoring food samples; Secondary data analysis / Previously collected data | Not specified | Alaska Native | United States | Not mentioned | Not mentioned |
| Gaudin 2014 | No | Secondary data analysis / Previously collected data; Quantitative observational study; Qualitative | Cree | First Nations | Canada | Not mentioned | Study results are expected to inform an specific broader strategy, program, or intervention to control exposure, improve environmental quality, or restore food system |
| Golzadeh 2020 | Yes | Biomonitoring food samples | Bigstone Cree Nation | First Nations | Canada | Research requested by Indigenous population; Community members participated conducting research activities; Research methods were designed in collaboration with Indigenous Population members; Results discussion were conducted in collaboration with Indigenous Population members | Study results are expected to inform an specific broader strategy, program, or intervention to control exposure, improve environmental quality, or restore food system |
| Golzadeh 2021 | Yes | Biomonitoring food samples | Bigstone Cree Nation | First Nations | Canada | Activity to receive community feedback; Community members participated conducting research activities; Research methods were designed in collaboration with Indigenous Population members | Not mentioned |
| Guidotti 2018 | No | Biomonitoring human samples; Biomonitoring food samples; Quantitative observational study | First Nations and Métis "(Aboriginal communities are not identified, in order to minimize harm.)" | First Nations; Metis | Canada | Information session conducted | Not mentioned |
| Guidotti 2018 | No | Biomonitoring human samples; Biomonitoring food samples | Not specified | First Nations; Metis | Canada | Information session conducted | Study results are already part of a broader strategy, program, or intervention to control exposure, improve environmental quality, or restore food system |
| Hacon 2020 | No | Biomonitoring food samples; Secondary data analysis / Previously collected data | Not specified | Amazon Indigenous Peoples | Brazil | Not mentioned | Not mentioned |
| Hahn 2022 | No | Biomonitoring food samples | Muckleshoot Tribe, Squaxin Island Tribe, Tulalip Tribes, Swinomish Indian Tribal Community, Lower Elwha Klallam Tribe, Suquamish Tribe, Squamish Nation, Stz’uminus First Nation, Cowichan Tribes, Tsartlip First Nation, Pauquachin First Nation, Songhees Nation, Esquimalt Nation, Pacheedaht First Nation, T’Sou-ke First Nation, Penela- kut Tribe, Qualicum Nation, and We Wai Kai Nation | First Nations; Native American | United States; Canada | Not mentioned | Not mentioned |
| Hardell 2010 | No | Biomonitoring food samples | Aleut | Circumpolar | United States | Community members participated conducting research activities | Not mentioned |
| Hlimi 2012 | No | Quantitative observational study | Cree First Nations | First Nations | Canada | Information session conducted; Activity to receive community feedback | Not mentioned |
| Hu 2017 | No | Secondary data analysis / Previously collected data; Quantitative observational study; Other: Simulation and modelling | Inuit | Circumpolar | Canada | Information session conducted; Activity to receive community feedback | Not mentioned |
| Irvine 2014 | No | Biomonitoring human samples; Biomonitoring food samples; Environmental monitoring; Quantitative observational study | Cold Lake First Nations | First Nations | Canada | Not mentioned | Not mentioned |
| Jacques 2024 | No | Biomonitoring human samples; Quantitative observational study; Demographics questionnaire | Yanomami | Amazon Indigenous Peoples | Brazil | Information session conducted; Community members participated conducting research activities | Not mentioned |
| Ji 2019 | No | Environmental monitoring | Nenets | Circumpolar | Russia | Not mentioned | Not mentioned |
| Johnson 2015 | No | Biomonitoring food samples; Environmental monitoring | Haisla First Nation | First Nations | Canada | Not mentioned | Study results are expected to inform an specific broader strategy, program, or intervention to control exposure, improve environmental quality, or restore food system |
| Johnson-Down 2015 | No | Biomonitoring human samples; Secondary data analysis / Previously collected data; Quantitative observational study | Cree (Eeyouch) | First Nations | Canada | Community members participated conducting research activities; Research methods were designed in collaboration with Indigenous Population members | Study results are already part of a broader strategy, program, or intervention to control exposure, improve environmental quality, or restore food system |
| Jordan-Ward 2022 | No | Biomonitoring food samples | Yupik | Circumpolar | United States | Not mentioned | Not mentioned |
| Jordan-Ward 2022 | No | Biomonitoring food samples | Yupik | Circumpolar | United States | Not mentioned | Not mentioned |
| Jordan-Ward 2024 | Yes | Biomonitoring food samples | Yupik | Circumpolar | United States | Not mentioned | Not mentioned |
| Juric 2017 | Yes | Biomonitoring human samples; Biomonitoring food samples; Secondary data analysis / Previously collected data; Quantitative observational study; Other: Simulation and modelling | First Nations (Ontario) | First Nations | Canada | Information session conducted; Activity to receive community feedback; Community members participated conducting research activities | Not mentioned |
| Juric 2018 | Yes | Biomonitoring food samples; Secondary data analysis / Previously collected data; Quantitative observational study; Other: Simulation and modelling | First Nations (Ontario) | First Nations | Canada | Information session conducted; Activity to receive community feedback; Community members participated conducting research activities; Research methods were designed in collaboration with Indigenous Population members | Study results are expected to inform an specific broader strategy, program, or intervention to control exposure, improve environmental quality, or restore food system |
| Kempton 2021 | No | Biomonitoring human samples; Secondary data analysis / Previously collected data; Quantitative observational study | Munduruku communities (SawréMuybu, SawréAboy, and Poxo Muybu) | Amazon Indigenous Peoples | Brazil | Information session conducted; Research requested by Indigenous population; Community members participated conducting research activities; Research methods were designed in collaboration with Indigenous Population members | Study results are expected to inform an specific broader strategy, program, or intervention to control exposure, improve environmental quality, or restore food system |
| Kenny 2019 | No | Biomonitoring human samples; Secondary data analysis / Previously collected data; Quantitative observational study | Inuit | Circumpolar | Canada | Community members participated conducting research activities; Results discussion were conducted in collaboration with Indigenous Population members | Not mentioned |
| KrarupHansen 2022 | No | Biomonitoring food samples; Indigenous knowledge and Western sciences were integrated to develop methods | Sámi | Circumpolar | Norway | Information session conducted; Activity to receive community feedback; Community members participated conducting research activities; Research methods were designed in collaboration with Indigenous Population members | Study results are expected to inform an specific broader strategy, program, or intervention to control exposure, improve environmental quality, or restore food system |
| Kwaansa-Ansah 2010 | No | Biomonitoring human samples; Demographics questionnaire | Not specified | Not specified | Ghana | Not mentioned | Not mentioned |
| Laffont 2011 | No | Biomonitoring human samples; Demographics questionnaire | Not specified | Amazon Indigenous Peoples | Bolivia | Not mentioned | Not mentioned |
| Laird 2013 | No | Biomonitoring food samples; Secondary data analysis / Previously collected data | First Nations | First Nations | Canada | Not mentioned | Not mentioned |
| Laird 2013 | No | Biomonitoring human samples; Biomonitoring food samples; Secondary data analysis / Previously collected data; Quantitative observational study | Inuit | First Nations | Canada | Results discussion were conducted in collaboration with Indigenous Population members | Study results are expected to inform an specific broader strategy, program, or intervention to control exposure, improve environmental quality, or restore food system |
| Laird 2018 | Yes | Biomonitoring food samples | First Nations (Dehcho Region) | First Nations | Canada | Not mentioned | Study results are already part of a broader strategy, program, or intervention to control exposure, improve environmental quality, or restore food system |
| Lavers 2013 | No | Biomonitoring food samples | Not specified | Aboriginal and Torres Strait Islander | Australia | Not mentioned | Not mentioned |
| Liberda 2011 | No | Biomonitoring human samples; Biomonitoring food samples; Quantitative observational study | Cree Nation of Oujé-Bougoumou, Fort Albany First Nation, and Weenusk First Nation | First Nations | Canada | Information session conducted; Community members participated conducting research activities | Not mentioned |
| Liberda 2014 | No | Biomonitoring human samples | Cree First Nations | First Nations | Canada | Activity to receive community feedback; Community members participated conducting research activities; Research methods were designed in collaboration with Indigenous Population members | Study results are expected to inform an specific broader strategy, program, or intervention to control exposure, improve environmental quality, or restore food system |
| Liberda 2018 | No | Biomonitoring human samples; Quantitative observational study | Cree | First Nations | Canada | Research requested by Indigenous population; Community members participated conducting research activities; Research methods were designed in collaboration with Indigenous Population members | Study results are expected to inform an specific broader strategy, program, or intervention to control exposure, improve environmental quality, or restore food system |
| Liddell 2022 | No | Qualitative | Gulf Coast Tribe | Native American | United States | Information session conducted; Activity to receive community feedback; Research requested by Indigenous population; Community members participated conducting research activities; Research methods were designed in collaboration with Indigenous Population members; Results discussion were conducted in collaboration with Indigenous Population members | Study results are expected to inform an specific broader strategy, program, or intervention to control exposure, improve environmental quality, or restore food system |
| Little 2019 | No | Biomonitoring human samples; Secondary data analysis / Previously collected data; Quantitative observational study | Inuit | Circumpolar | Canada | Research requested by Indigenous population; Results discussion were conducted in collaboration with Indigenous Population members | Study results are expected to inform an specific broader strategy, program, or intervention to control exposure, improve environmental quality, or restore food system |
| Lucier 2023 | Yes | Environmental monitoring; Qualitative; Indigenous research methods; Indigenous knowledge and Western sciences were integrated to develop methods | Wauzhushk Onigum | First Nations | Canada | Information session conducted; Activity to receive community feedback; Research requested by Indigenous population; Community members participated conducting research activities; Research methods were designed in collaboration with Indigenous Population members; Results discussion were conducted in collaboration with Indigenous Population members | Study results are already part of a broader strategy, program, or intervention to control exposure, improve environmental quality, or restore food system |
| Makarov 2018 | No | Biomonitoring food samples | Not specified | Circumpolar | Russia | Not mentioned | Not mentioned |
| Martinez-Levasseur 2020 | Yes | Biomonitoring food samples; Qualitative ; Indigenous knowledge and Western sciences were integrated to develop methods | Inuit | Circumpolar | Canada | Activity to receive community feedback; Research requested by Indigenous population; Community members participated conducting research activities; Research methods were designed in collaboration with Indigenous Population members; Results discussion were conducted in collaboration with Indigenous Population members | Study results are already part of a broader strategy, program, or intervention to control exposure, improve environmental quality, or restore food system |
| Marushka 2017 | Yes | Biomonitoring food samples; Secondary data analysis / Previously collected data; Quantitative observational study | First Nations | First Nations | Canada | Information session conducted; Activity to receive community feedback; Community members participated conducting research activities | Not mentioned |
| Marushka 2018 | Yes | Biomonitoring food samples; Secondary data analysis / Previously collected data; Quantitative observational study | First Nations (BC) | First Nations | Canada | Information session conducted; Activity to receive community feedback; Community members participated conducting research activities | Not mentioned |
| Marushka 2018 | Yes | Biomonitoring food samples; Secondary data analysis / Previously collected data; Quantitative observational study | First Nations (Ontario and Manitoba) | First Nations | Canada | Information session conducted; Activity to receive community feedback; Community members participated conducting research activities | Not mentioned |
| Marushka 2021 | Yes | Biomonitoring food samples; Secondary data analysis / Previously collected data; Quantitative observational study | First Nations | First Nations | Canada | Not mentioned | Study results are expected to inform an specific broader strategy, program, or intervention to control exposure, improve environmental quality, or restore food system |
| Marushka 2024 | Yes | Biomonitoring human samples; Secondary data analysis / Previously collected data; Quantitative observational study; Demographics questionnaire | Hagwilget, Kitsumkalum, Skidegate, Nuxalk, 'Namgis, Tla'amin | First Nations | Canada | Not mentioned | Not mentioned |
| Matwee 2019 | No | Biomonitoring food samples | Montreal Lake Cree Nation | First Nations | Canada | Community members participated conducting research activities | Not mentioned |
| Maurice 2021 | No | Biomonitoring human samples; Biomonitoring food samples; Environmental monitoring; Quantitative observational study | Wayãpi Indians | Amazon Indigenous Peoples | French Guiana | Not mentioned | Not mentioned |
| Mayor 2024 | No | Biomonitoring food samples | Achuar, Kichwa, Yagua | Amazon Indigenous Peoples | Peru | Community members participated conducting research activities | Not mentioned |
| McAuley 2018 | Yes | Biomonitoring food samples | Cold Lake First Nations, Swan River First Nations, Chipewyan Prairie Déné First Nation | First Nations | Canada | Activity to receive community feedback; Research requested by Indigenous population; Community members participated conducting research activities; Research methods were designed in collaboration with Indigenous Population members | Study results are expected to inform an specific broader strategy, program, or intervention to control exposure, improve environmental quality, or restore food system |
| Medehouenou 2010 | No | Biomonitoring human samples; Quantitative observational study | Nunavik Inuit | Circumpolar | Canada | Activity to receive community feedback | Not mentioned |
| Mergler 2023 | Yes | Biomonitoring human samples; Secondary data analysis / Previously collected data; Quantitative observational study; Demographics questionnaire | Grassy Narrows First Nation | First Nations | Canada | Information session conducted; Activity to receive community feedback; Community members participated conducting research activities; Research methods were designed in collaboration with Indigenous Population members; Results discussion were conducted in collaboration with Indigenous Population members | Study results are already part of a broader strategy, program, or intervention to control exposure, improve environmental quality, or restore food system |
| Minick 2019 | No | Biomonitoring food samples; Environmental monitoring | Native American Tribes (Requested to remain anonymous) | Native American | United States | Activity to receive community feedback; Community members participated conducting research activities; Research methods were designed in collaboration with Indigenous Population members; Results discussion were conducted in collaboration with Indigenous Population members | Not mentioned |
| Mnisi 2017 | No | Biomonitoring food samples | Swazi | Swazi | Swaziland | Not mentioned | Not mentioned |
| Moriarity 2020 | No | Biomonitoring human samples; Biomonitoring food samples; Secondary data analysis / Previously collected data; Quantitative observational study | Eeyou Istchee (Cree) | First Nations | Canada | Activity to receive community feedback; Results discussion were conducted in collaboration with Indigenous Population members | Study results are expected to inform an specific broader strategy, program, or intervention to control exposure, improve environmental quality, or restore food system |
| Moriarity 2020 | No | Biomonitoring human samples; Biomonitoring food samples; Secondary data analysis / Previously collected data; Quantitative observational study | Eeyou Istchee (Cree) | First Nations | Canada | Activity to receive community feedback; Results discussion were conducted in collaboration with Indigenous Population members | Study results are expected to inform an specific broader strategy, program, or intervention to control exposure, improve environmental quality, or restore food system |
| Moriarity 2020 | No | Environmental monitoring | Fort Albany First Nation (Mushkegowuk Cree) | First Nations | Canada | Activity to receive community feedback; Community members participated conducting research activities; Research methods were designed in collaboration with Indigenous Population members | Study results are expected to inform an specific broader strategy, program, or intervention to control exposure, improve environmental quality, or restore food system |
| Moriarity 2021 | No | Secondary data analysis / Previously collected data; Quantitative observational study | Eeyouch (Cree First Nation) | First Nations | Canada | Activity to receive community feedback; Research methods were designed in collaboration with Indigenous Population members; Results discussion were conducted in collaboration with Indigenous Population members | Study results are already part of a broader strategy, program, or intervention to control exposure, improve environmental quality, or restore food system |
| Moriarity 2023 | No | Biomonitoring food samples | Cree of Whapmagoostui, Chisasibi, Wemindji, Eastmain, Waskaganish, Nemaska, Waswanipi, Ouje-Bougoumou, Mistissini First Nations | First Nations | Canada | Community members participated conducting research activities | Not mentioned |
| Moriarity 2024 | Yes | Biomonitoring food samples | Moose Cree First Nation (Moose Factory), Fort Albany First Nation, Kashechewan First Nation, Attawapiskat First Nation, Peawanuck First Nation | First Nations | Canada | Community members participated conducting research activities | Study results are expected to inform an specific broader strategy, program, or intervention to control exposure, improve environmental quality, or restore food system |
| Newman 2014 | Yes | Biomonitoring human samples; Secondary data analysis / Previously collected data; Quantitative observational study | Mohawk Nation of Akwesasne | First Nations; Native American | United States; Canada | Activity to receive community feedback; Research requested by Indigenous population; Community members participated conducting research activities; Results discussion were conducted in collaboration with Indigenous Population members | Study results are already part of a broader strategy, program, or intervention to control exposure, improve environmental quality, or restore food system |
| Nieboer 2017 | Yes | Biomonitoring human samples; Quantitative observational study | Eeyou (Cree First Nation) | First Nations | Canada | Activity to receive community feedback; Community members participated conducting research activities | Not mentioned |
| O'Callaghan-Gordo 2021 | No | Biomonitoring human samples; Secondary data analysis / Previously collected data; Quantitative observational study | Achuar, Quechua, Kichwa, Kukama, and other peoples | Amazon Indigenous Peoples | Peru | Activity to receive community feedback; Community members participated conducting research activities | Study results are expected to inform an specific broader strategy, program, or intervention to control exposure, improve environmental quality, or restore food system |
| Olivero-Verbel 2016 | No | Biomonitoring human samples; Biomonitoring food samples; Quantitative observational study | Amazon indigenous | Amazon Indigenous Peoples | Colombia | Information session conducted | Not mentioned |
| Orta-Martínez 2018 | No | Environmental monitoring | Amazon Indigenous | Amazon Indigenous Peoples | Peru | Community members participated conducting research activities | Not mentioned |
| Packull-McCormick 2023 | Yes | Biomonitoring food samples | First Nations | First Nations | Canada | Not mentioned | Not mentioned |
| Padhan 2018 | No | Biomonitoring food samples | tribal people of Koraput | Tribal people of Koraput | India | Not mentioned | Not mentioned |
| Panduro 2020 | No | Biomonitoring food samples; Quantitative observational study | Santa Rosa de Tamaya and Tipishca | Amazon Indigenous Peoples | Peru | Not mentioned | Not mentioned |
| Pang 2016 | No | Secondary data analysis / Previously collected data; Quantitative observational study | American Indian (Communities requested to remain anonymous) | Native American | United States | Activity to receive community feedback | Not mentioned |
| Paunescu 2013 | No | Biomonitoring human samples; Quantitative observational study | Eeyou (Cree First Nations) | First Nations | Canada | Community members participated conducting research activities | Not mentioned |
| Paunescu 2013 | No | Biomonitoring human samples; Quantitative observational study | Inuit | Circumpolar | Canada | Not mentioned | Study results are expected to inform an specific broader strategy, program, or intervention to control exposure, improve environmental quality, or restore food system |
| Peplow 2014 | Yes | Biomonitoring human samples; Quantitative observational study | Wayana | Amazon Indigenous Peoples | Suriname | Activity to receive community feedback; Research requested by Indigenous population; Community members participated conducting research activities; Research methods were designed in collaboration with Indigenous Population members; Results discussion were conducted in collaboration with Indigenous Population members | Study results are already part of a broader strategy, program, or intervention to control exposure, improve environmental quality, or restore food system |
| Perini 2021 | No | Biomonitoring human samples; Quantitative observational study | Munduruku | Amazon Indigenous Peoples | Brazil | Information session conducted; Community members participated conducting research activities | Not mentioned |
| Philibert 2020 | No | Biomonitoring human samples; Secondary data analysis / Previously collected data; Quantitative observational study | The Asubpeeschoseewagong Netum Anishinabek (Grassy Narrows First Nation) | First Nations | Canada | Information session conducted; Activity to receive community feedback; Research requested by Indigenous population; Community members participated conducting research activities; Research methods were designed in collaboration with Indigenous Population members; Results discussion were conducted in collaboration with Indigenous Population members | Study results are expected to inform an specific broader strategy, program, or intervention to control exposure, improve environmental quality, or restore food system |
| Philibert 2022 | Yes | Biomonitoring human samples; Quantitative observational study | Asubpeeschoseewagong Netum Anishinabek (Grassy Narrows First Nation) | First Nations | Canada | Activity to receive community feedback; Research requested by Indigenous population; Community members participated conducting research activities; Research methods were designed in collaboration with Indigenous Population members; Results discussion were conducted in collaboration with Indigenous Population members | Study results are expected to inform an specific broader strategy, program, or intervention to control exposure, improve environmental quality, or restore food system |
| Phillips 2014 | No | Biomonitoring food samples; Environmental monitoring; Quantitative observational study | Te Arawa tribe (Māori) | Pacific Islander | New Zealand | Information session conducted; Community members participated conducting research activities; Research methods were designed in collaboration with Indigenous Population members | Not mentioned |
| Pontual 2021 | Yes | Biomonitoring human samples; Quantitative observational study | Inuit (Nunavit) | Circumpolar | Canada | Information session conducted; Community members participated conducting research activities; Research methods were designed in collaboration with Indigenous Population members | Not mentioned |
| Ratelle 2018 | No | Biomonitoring human samples; Quantitative observational study; Qualitative | Deh Gah Gotie; West Point; Jean Marie River; Ka’a’geeTu; Sambaa Ke, Katlodeech (Dene First Nation) | First Nations | Canada | Information session conducted; Community members participated conducting research activities | Study results are expected to inform an specific broader strategy, program, or intervention to control exposure, improve environmental quality, or restore food system |
| Ratelle 2018 | No | Biomonitoring human samples; Quantitative observational study; Qualitative ; Indigenous knowledge and Western sciences were integrated to develop methods | Dehcho First Nations | First Nations | Canada | Information session conducted; Activity to receive community feedback; Research requested by Indigenous population; Community members participated conducting research activities; Research methods were designed in collaboration with Indigenous Population members | Study results are already part of a broader strategy, program, or intervention to control exposure, improve environmental quality, or restore food system |
| Ratelle 2018 | No | Biomonitoring human samples; Quantitative observational study | Dene and Métis (Northwest Territories Mackenzie Valley) | First Nations; Metis | Canada | Information session conducted; Activity to receive community feedback; Community members participated conducting research activities | Study results are already part of a broader strategy, program, or intervention to control exposure, improve environmental quality, or restore food system |
| Ratelle 2020 | No | Biomonitoring human samples; Quantitative observational study | Dehcho and Sahtú First Nations | First Nations | Canada | Information session conducted; Activity to receive community feedback; Community members participated conducting research activities; Research methods were designed in collaboration with Indigenous Population members | Study results are already part of a broader strategy, program, or intervention to control exposure, improve environmental quality, or restore food system |
| Reiner 2016 | No | Biomonitoring food samples; Secondary data analysis / Previously collected data | Aleut Community | Circumpolar | United States | Community members participated conducting research activities | Not mentioned |
| Reyes 2015 | No | Environmental monitoring | Fort Albany First Nation | First Nations | Canada | Not mentioned | Not mentioned |
| Ripley 2018 | No | Biomonitoring human samples; Secondary data analysis / Previously collected data; Quantitative observational study | Cree First Nations of Eeyou Istchee | First Nations | Canada | Not mentioned | Not mentioned |
| Rivera 2016 | No | Biomonitoring food samples | Tacana | Amazon Indigenous Peoples | Bolivia | Community members participated conducting research activities | Not mentioned |
| Rosell-Melé 2018 | No | Environmental monitoring | Not specified | Amazon Indigenous Peoples | Peru | Activity to receive community feedback; Community members participated conducting research activities | Not mentioned |
| Russell 2015 | No | Biomonitoring food samples; Quantitative observational study; Qualitative | Gumbaynggirr Aboriginal community | Aboriginal and Torres Strait Islander | Australia | Information session conducted; Activity to receive community feedback; Results discussion were conducted in collaboration with Indigenous Population members | Study results are expected to inform an specific broader strategy, program, or intervention to control exposure, improve environmental quality, or restore food system |
| Samuel-Nakamura 2017 | No | Biomonitoring food samples; Environmental monitoring; Quantitative observational study | Diné (Navajo) | Native American | United States | Not mentioned | Not mentioned |
| Sarkar 2019 | Yes | Biomonitoring food samples; Environmental monitoring | Makkovik Community (Inuit) | Circumpolar | Canada | Activity to receive community feedback; Research requested by Indigenous population; Community members participated conducting research activities | Study results are expected to inform an specific broader strategy, program, or intervention to control exposure, improve environmental quality, or restore food system |
| Scammell 2020 | Yes | Biomonitoring human samples | Navajo | Native American | United States | Community members participated conducting research activities | Not mentioned |
| Schartup 2015 | No | Environmental monitoring | Inuit (Nunatsiavut, Labrador) | Circumpolar | Canada | Not mentioned | Not mentioned |
| Schuster 2011 | Yes | Biomonitoring food samples; Quantitative observational study | Vuntut Gwitchin First Nation | First Nations | Canada | Activity to receive community feedback; Community members participated conducting research activities; Results discussion were conducted in collaboration with Indigenous Population members | Study results are expected to inform an specific broader strategy, program, or intervention to control exposure, improve environmental quality, or restore food system |
| Seabert 2014 | No | Biomonitoring human samples; Quantitative observational study | Wapekeka First Nation and Kasa-bonika Lake First Nation | First Nations | Canada | Activity to receive community feedback; Community members participated conducting research activities; Research methods were designed in collaboration with Indigenous Population members | Not mentioned |
| Silva 2024 | No | Biomonitoring human samples; Quantitative observational study; Demographics questionnaire | Munduruku | Amazon Indigenous Peoples | Brazil | Information session conducted; Activity to receive community feedback; Community members participated conducting research activities | Study results are expected to inform an specific broader strategy, program, or intervention to control exposure, improve environmental quality, or restore food system |
| Simon 2020 | No | Environmental monitoring; Secondary data analysis / Previously collected data; Qualitative | No specified | Native American; Alaska Native; Hawaiian | United States | Not mentioned | Not mentioned |
| Simpson 2024 | Yes | Biomonitoring human samples; Secondary data analysis / Previously collected data; Quantitative observational study | Vuntut Gwitchin First Nation | First Nations | Canada | Information session conducted; Activity to receive community feedback; Community members participated conducting research activities | Study results are expected to inform an specific broader strategy, program, or intervention to control exposure, improve environmental quality, or restore food system |
| Skalny 2019 | No | Biomonitoring human samples; Quantitative observational study | Amis | Amis | Taiwan | Not mentioned | Not mentioned |
| Smith 2023 | No | Biomonitoring food samples | Confederated Tribes of Warm Springs | Native American | United States | Community members participated conducting research activities | Not mentioned |
| Sobolev 2021 | No | Biomonitoring human samples | Nenets | Circumpolar | Russia | Information session conducted | Not mentioned |
| SomnathBhowmik 2012 | No | Biomonitoring food samples | Not specified | Not specified | India | Not mentioned | Not mentioned |
| Sonne 2023 | No | Biomonitoring human samples; Biomonitoring food samples; Quantitative observational study; Qualitative; Demographics questionnaire | Inuit | Circumpolar | Greenland | Community members participated conducting research activities | Not mentioned |
| Sorokina 2022 | No | Biomonitoring human samples; Quantitative observational study; Demographics questionnaire | Nenets (Samoyed) | Circumpolar | Russia | Not mentioned | Not mentioned |
| Souza-Araujo 2016 | No | Biomonitoring food samples | Xikrin | Amazon Indigenous Peoples | Brazil | Community members participated conducting research activities | Not mentioned |
| St 2015 | No | Environmental monitoring | Not specified | Circumpolar; First Nations; Metis | Canada | Not mentioned | Not mentioned |
| Stachiw 2019 | No | Biomonitoring food samples | Not specified | First Nations; Metis | Canada | Not mentioned | Not mentioned |
| Stewart 2011 | No | Biomonitoring food samples; Quantitative observational study | Māori | Pacific Islander | New Zealand | Activity to receive community feedback; Community members participated conducting research activities; Research methods were designed in collaboration with Indigenous Population members | Not mentioned |
| Stroink 2012 | No | Quantitative observational study; Qualitative | Ginoogaming First Nation, Aroland First Nation, and Eabametoong First Nation | First Nations | Canada | Activity to receive community feedback; Research requested by Indigenous population; Community members participated conducting research activities; Research methods were designed in collaboration with Indigenous Population members | Study results are expected to inform an specific broader strategy, program, or intervention to control exposure, improve environmental quality, or restore food system |
| Suami 2019 | No | Biomonitoring food samples | Not specified | Not specified | Democratic Republic of the Congo | Not mentioned | Not mentioned |
| Tam 2015 | No | Biomonitoring human samples; Secondary data analysis / Previously collected data; Quantitative observational study | Eeyou Istchee (Cree First Nation) | First Nations | Canada | Community members participated conducting research activities | Study results are expected to inform an specific broader strategy, program, or intervention to control exposure, improve environmental quality, or restore food system |
| Tanamal 2021 | No | Biomonitoring food samples; Quantitative observational study | Dene First Nation | First Nations | Canada | Community members participated conducting research activities | Not mentioned |
| Thompson 2017 | Yes | Biomonitoring food samples; Indigenous knowledge and Western sciences were integrated to develop methods | Gitga'at First Nation | First Nations | Canada | Activity to receive community feedback; Research requested by Indigenous population; Community members participated conducting research activities; Research methods were designed in collaboration with Indigenous Population members | Study results are already part of a broader strategy, program, or intervention to control exposure, improve environmental quality, or restore food system |
| Tian 2011 | Yes | Biomonitoring human samples; Biomonitoring food samples; Secondary data analysis / Previously collected data; Quantitative observational study | Inuit | Circumpolar | Canada | Activity to receive community feedback; Community members participated conducting research activities; Research methods were designed in collaboration with Indigenous Population members | Not mentioned |
| Tikhonov 2021 | Yes | Biomonitoring human samples; Secondary data analysis / Previously collected data; Quantitative observational study | First Nations (Canada, on reserve) | First Nations | Canada | Information session conducted; Activity to receive community feedback; Research requested by Indigenous population; Community members participated conducting research activities; Research methods were designed in collaboration with Indigenous Population members; Results discussion were conducted in collaboration with Indigenous Population members | Study results are expected to inform an specific broader strategy, program, or intervention to control exposure, improve environmental quality, or restore food system |
| Trukhin 2018 | No | Biomonitoring food samples | Chukchi | Circumpolar | Russia | Community members participated conducting research activities | Not mentioned |
| Unguryanu 2023 | No | Biomonitoring food samples; Secondary data analysis / Previously collected data | Nenets Autonomous Okrug | Circumpolar | Russia | Not mentioned | Not mentioned |
| Valdelamar-Villegas 2020 | No | Biomonitoring human samples; Quantitative observational study | Amazonian Indigenous populations (Apaporis River basin) | Amazon Indigenous Peoples | Colombia | Not mentioned | Not mentioned |
| Valera 2011 | No | Biomonitoring human samples; Secondary data analysis / Previously collected data; Quantitative observational study | Cree First Nations | First Nations | Canada | Community members participated conducting research activities | Study results are expected to inform an specific broader strategy, program, or intervention to control exposure, improve environmental quality, or restore food system |
| Valera 2013 | No | Biomonitoring human samples; Secondary data analysis / Previously collected data; Quantitative observational study | Inuit | Circumpolar | Canada | Information session conducted | Not mentioned |
| Valera 2013 | No | Biomonitoring human samples; Quantitative observational study | Inuit | Circumpolar | Greenland | Information session conducted; Community members participated conducting research activities | Not mentioned |
| VanHorne 2024 | Yes | Environmental monitoring; Secondary data analysis / Previously collected data; Indigenous knowledge and Western sciences were integrated to develop methods; Demographics questionnaire | Diné | Native American | United States | Information session conducted; Activity to receive community feedback; Community members participated conducting research activities; Research methods were designed in collaboration with Indigenous Population members; Results discussion were conducted in collaboration with Indigenous Population members | Study results are already part of a broader strategy, program, or intervention to control exposure, improve environmental quality, or restore food system |
| Varakina 2022 | No | Biomonitoring human samples | Nenets | Circumpolar | Russia | Not mentioned | Not mentioned |
| Varty 2021 | No | Environmental monitoring | Not specified (Nunavut) | Circumpolar | Canada | Not mentioned | Not mentioned |
| Vega 2018 | No | Biomonitoring human samples; Quantitative observational study | Yanomami | Amazon Indigenous Peoples | Brazil | Information session conducted; Research requested by Indigenous population; Community members participated conducting research activities | Not mentioned |
| VelásquezRamírez 2021 | No | Environmental monitoring | San Jacinto Native Community and Kotzimba Native Community | Amazon Indigenous Peoples | Peru | Not mentioned | Study results are expected to inform an specific broader strategy, program, or intervention to control exposure, improve environmental quality, or restore food system |
| vonHippel 2018 | Yes | Biomonitoring food samples | Yupik | Circumpolar | United States | Research requested by Indigenous population | Not mentioned |
| Walker 2020 | Yes | Biomonitoring food samples; Qualitative ; Indigenous knowledge and Western sciences were integrated to develop methods | Inuit (Nunavut) | Circumpolar | Canada | Activity to receive community feedback; Research requested by Indigenous population; Community members participated conducting research activities; Research methods were designed in collaboration with Indigenous Population members | Study results are expected to inform an specific broader strategy, program, or intervention to control exposure, improve environmental quality, or restore food system |
| Watson 2020 | No | Biomonitoring human samples;Secondary data analysis / Previously collected data; Quantitative observational study | Aishalton, Karaudarnau, Parabara, Shulinab, Kuyuwini | Amazon Indigenous Peoples | Guyana | Activity to receive community feedback; Research requested by Indigenous population | Not mentioned |
| Webb 2016 | No | Biomonitoring human samples; Quantitative observational study | Kichwa, Achuar and Urarina Nations | Amazon Indigenous Peoples | Peru; Ecuador | Information session conducted | Not mentioned |
| Weinhouse 2020 | No | Biomonitoring human samples; Quantitative observational study | Native communities (Amarakaeri Communal Reserve) | Amazon Indigenous Peoples | Peru | Not mentioned | Not mentioned |
| Wyatt 2019 | No | Biomonitoring human samples; Secondary data analysis / Previously collected data; Quantitative observational study | Boca Isiriwe, Diamante, Isla de los Valles, Masenawa,Palotoa Teparo, Puerto Azul, Puerto Luz, Queros, San Lorenzo, Shintuya, Shipetiari | Amazon Indigenous Peoples | Peru | Not mentioned | Not mentioned |
