# Supplementary material for: Community engagement in Indigenous food systems contamination studies: A systematic scoping review
Source: PLoS One. 2025 Nov 14;20(11):e0336439. doi: 10.1371/journal.pone.0336439 (PMC12617898; doi:10.1371/journal.pone.0336439)
Supplement: S3 Table — (DOCX) [file pone.0336439.s003.docx]

| **Indigenous Population** | **Frequency** |
| --- | --- |
| Inuit | 24 |
| Cree First Nations | 9 |
| Anishinaabe | 8 |
| Yupik | 8 |
| First Nations | 7 |
| Dene | 4 |
| Eeyou Istchee (Cree First Nation) | 4 |
| Achuar | 3 |
| Chukchi | 3 |
| Cold Lake First Nations | 3 |
| Fort Albany First Nation | 3 |
| Kichwa | 3 |
| Māori | 3 |
| Munduruku communities | 3 |
| Nenets | 3 |
| Nunavimmiut | 3 |
| Vuntut Gwitchin First Nation | 3 |
| Yanomami | 3 |
| Bigstone Cree Nation | 2 |
| Boca Isiriwe | 2 |
| Deh Gah Gotie First Nation | 2 |
| Dehcho First Nations | 2 |
| Diamante | 2 |
| Eeyou (Cree First Nation) | 2 |
| First Nations (Ontario) | 2 |
| Grassy Narrows First Nation (Asubpeeschoseewagong Netum Anishinabek) | 2 |
| Inuit (Nunavut) | 2 |
| Jean Marie River First Nation | 2 |
| Ka’a’gee Tu First Nation | 2 |
| Masenawa | 2 |
| Metis | 2 |
| Métis | 2 |
| Palotoa Teparo | 2 |
| Poxo Muybu (Munduruku communities) | 2 |
| Puerto Azul | 2 |
| Puerto Luz | 2 |
| Queros | 2 |
| San Lorenzo | 2 |
| Sawré Aboy (Munduruku communities) | 2 |
| Sawré Muybu (Munduruku communities) | 2 |
| Shintuya | 2 |
| Shipetiari | 2 |
| West Point First Nation | 2 |
| Xeni Gwet'in First Nation | 2 |
| 'Namgis, Tla'amin | 1 |
| Aishalton | 1 |
| Aklavik | 1 |
| Akwesasne Mohawk Nation | 1 |
| Aleut | 1 |
| Aleut Community | 1 |
| Amarakaeri Communal Reserve | 1 |
| Amazon Indigenous | 1 |
| Amazon Indigenous Peoples | 1 |
| Amazonian Indigenous populations (Apaporis River basin) | 1 |
| American Indian (Communities requested to remain anonymous) | 1 |
| Amis | 1 |
| Aroland First Nation | 1 |
| Attawapiskat First Nation | 1 |
| Aymara-Quechua | 1 |
| Bapedi | 1 |
| Boca Manu | 1 |
| Bora | 1 |
| Chipewyan Prairie Déné First Nation | 1 |
| Chisasibi | 1 |
| Confederated Tribes of the Umatilla Indian Reservation | 1 |
| Confederated Tribes of Warm Springs | 1 |
| Cowichan Tribes | 1 |
| Cree Nation of Oujé-Bougoumou | 1 |
| Cree of Whapmagoostui | 1 |
| Dene First Nation | 1 |
| Diné | 1 |
| Diné (Navajo) | 1 |
| Eabametoong First Nation | 1 |
| Eastmain | 1 |
| Eeyou Istchee (Cree) | 1 |
| Eeyouch (Cree First Nation) | 1 |
| Esquimalt Nation | 1 |
| Esse Ejjas | 1 |
| First Nations (BC) | 1 |
| First Nations (Canada, on reserve) | 1 |
| First Nations (Dehcho Region) | 1 |
| First Nations (Ontario and Manitoba) | 1 |
| First Nations British Columbia | 1 |
| Fort Albany First Nation (Mushkegowuk Cree) | 1 |
| Fort Mcpherson | 1 |
| Ginoogaming First Nation | 1 |
| Gitga'at First Nation | 1 |
| Grand Portage Band of Lake Superior Chippewa (Chippewa) | 1 |
| Grassy Narrows First Nation | 1 |
| Gulf Coast Tribe | 1 |
| Gumbaynggirr Aboriginal community | 1 |
| Gwich’in | 1 |
| Gwitch'in | 1 |
| Hagwilget | 1 |
| Haisla First Nation | 1 |
| Inuit (Makkovik Community) | 1 |
| Inuit (Nunatsiavut, Labrador) | 1 |
| Inuit (Nunavik) | 1 |
| Inupiaq | 1 |
| Inupiaq (Selawik) | 1 |
| Inupiat | 1 |
| Inuvialuit | 1 |
| Inuvik | 1 |
| Isla de los Valles | 1 |
| Karaudarnau | 1 |
| Kasa-bonika Lake First Nation | 1 |
| Kashechewan First Nation | 1 |
| Katlodeech First Nation | 1 |
| Kayapó | 1 |
| Kitsumkalum | 1 |
| Kotzimba Native Community | 1 |
| Kukama | 1 |
| Kuyuwini | 1 |
| Lower Elwha Klallam Tribe | 1 |
| Lumbee Tribe | 1 |
| Mapuche-Huichille | 1 |
| Métis (Northwest Territories Mackenzie Valley) | 1 |
| Mistissini First Nations | 1 |
| Mohawk Nation | 1 |
| Mohawk Nation of Akwesasne | 1 |
| Montreal Lake Cree Nation | 1 |
| Moose Cree First Nation (Moose Factory) | 1 |
| Muckleshoot Tribe | 1 |
| Munduruku | 1 |
| Native American Tribes (Requested to remain anonymous) | 1 |
| Navajo | 1 |
| Nemaska | 1 |
| Nenets (Samoyed) | 1 |
| Nenets Autonomous Okrug | 1 |
| Nuxalk | 1 |
| Ocaina | 1 |
| Old Crow First Nation | 1 |
| Ouje-Bougoumou | 1 |
| Pacheedaht First Nation | 1 |
| Parabara | 1 |
| Pauquachin First Nation | 1 |
| Peawanuck First Nation | 1 |
| Penelakut Tribe | 1 |
| Qawalangin Tribe | 1 |
| Qualicum Nation | 1 |
| Quechua | 1 |
| Rabhas | 1 |
| Sahtú First Nations | 1 |
| Sambaa K’e First Nation | 1 |
| Sambaa Ke First Nation | 1 |
| Sámi | 1 |
| San Jacinto Native Community | 1 |
| Santa Rosa de Tamaya | 1 |
| Shulinab | 1 |
| Sivuqaq Yupik | 1 |
| Skidegate | 1 |
| Songhees Nation | 1 |
| Sonowal Kacharis | 1 |
| Squamish Nation | 1 |
| Squaxin Island Tribe | 1 |
| St. Lawrence Island Yupik | 1 |
| Stz’uminus First Nation | 1 |
| Suquamish Tribe | 1 |
| Swan River First Nation | 1 |
| Swazi | 1 |
| Swinomish Indian Tribal Community | 1 |
| T’Sou-ke First Nation | 1 |
| Tacana | 1 |
| Takanas | 1 |
| Te Arawa Tribe (Māori) | 1 |
| Tikuna | 1 |
| Tipishca | 1 |
| Tres Islas | 1 |
| Tribal people of Koraput | 1 |
| Tsartlip First Nation | 1 |
| Tsiigehtchic | 1 |
| Tsleil-Waututh Nation | 1 |
| Tulalip Tribes | 1 |
| Tupari | 1 |
| Uitoto | 1 |
| Urarina | 1 |
| Uru | 1 |
| Wapekeka First Nation | 1 |
| Waskaganish | 1 |
| Waswanipi | 1 |
| Wauzhushk Onigum | 1 |
| Wayana | 1 |
| Wayãpi | 1 |
| We Wai Kai Nation | 1 |
| Weenusk First Nation | 1 |
| Wemindji | 1 |
| Xikrin | 1 |
| Yagua | 1 |
| Yukon and K’atl’odeeche First Nation | 1 |
| Not specified | 21 |
