# Supplementary material for: Community engagement in Indigenous food systems contamination studies: A systematic scoping review
Source: PLoS One. 2025 Nov 14;20(11):e0336439. doi: 10.1371/journal.pone.0336439 (PMC12617898; doi:10.1371/journal.pone.0336439)
Supplement: Attachment — Submitted filename: Response2Reviewers_Sept29.docx [file pone.0336439.s005.docx]

**Response to reviewers PLOS ONE - Community engagement in Indigenous food systems contamination studies: A systematic scoping review**

**Editor’s response**

*As acknowledged by the reviewers, this research addresses important questions that will	contribute meaningfully to the field.*

*Both reviewers provided excellent suggestions to strengthen the manuscript by ensuring	methods are fully described and that conclusions are presented clearly and are supported	by the data. For acceptance, please revise the manuscript to address the following:*

*- describe methods and their implications in more detail.*

*- highlight key patterns and what these mean when describing results.*

*- ensure conclusions are supported by the data or clearly indicate how/when you	are relating your findings to existing literature.*

Thanks for your positive feedback and opportunity to revise our manuscript. We addressed each of the Reviewers’ comments in this document, with a particular focus on the key revisions you highlighted.

**Journal requirements**

*1.Please ensure that your manuscript meets PLOS ONE's style requirements, including those for file naming. The PLOS ONE style templates can be found at*

[*https://journals.plos.org/*](https://journals.plos.org/)

Thanks for the reminder. We are making every effort for the manuscript to meet the journal’s style.

*2. We note that your Data Availability Statement is currently as follows: All relevant data are within the manuscript and in Supporting Information files.*

*Please confirm at this time whether or not your submission contains all raw data required to replicate the results of your study. Authors must share the “minimal data set” for their submission. PLOS defines the minimal data set to consist of the data required to replicate all study findings reported in the article, as well as related metadata and methods (*[*https://journals.plos.org/*](../RR_1_CoAuthors_Comments/_blank)*).*

*For example, authors should submit the following data:*

*- The values behind the means, standard deviations and other measures reported;*

*- The values used to build graphs;*

*- The points extracted from images for analysis.*

*Authors do not need to submit their entire data set if only a portion of the data was used in the reported study.*

*If your submission does not contain these data, please either upload them as Supporting Information files or deposit them to a stable, public repository and provide us with the relevant URLs, DOIs, or accession numbers. For a list of recommended repositories, please see* [*https://journals.plos.org/*](https://journals.plos.org/plosone/s/recommended-repositories)*.*

*If there are ethical or legal restrictions on sharing a de-identified data set, please explain them in detail (e.g., data contain potentially sensitive information, data are owned by a third-party organization, etc.) and who has imposed them (e.g., an ethics committee). Please also provide contact information for a data access committee, ethics committee, or other institutional body to which data requests may be sent. If data are owned by a third party, please indicate how others may request data access*.

Thanks, we confirm that the current submission contains the minimal data set necessary to reproduce our results. The review data was uploaded as a supplementary material.

*3. Please upload a copy of Supporting Information 1 and 2 which you refer to in your text on page 37.*

We apologies for missing uploading the supporting information in our previous submission. We have now included the supporting information.

*4. If the reviewer comments include a recommendation to cite specific previously published works, please review and evaluate these publications to determine whether they are relevant and should be cited. There is no requirement to cite these works unless the editor has indicated otherwise.*

Noted

*5. Please review your reference list to ensure that it is complete and correct. If you have cited papers that have been retracted, please include the rationale for doing so in the manuscript text, or remove these references and replace them with relevant current references. Any changes to the reference list should be mentioned in the rebuttal letter that accompanies your revised manuscript. If you need to cite a retracted article, indicate the article’s retracted status in the References list and also include a citation and full reference for the retraction notice.*

**Reviewer #1:**

*This is an interesting piece of work. It’s clearly written and engages with a critical set of questions around Indigenous engagement in environmental health research. I appreciate the effort to bring visibility to issues surrounding decolonizing research practices in a structured way. That said, there are several areas that need revision to strengthen the manuscript and ensure the conclusions are well-supported by the data.*

Thank you for your review and positive comments. Below, we addressed each of the areas of improvement you highlighted.

***Areas for Revision:***

*1. The absence of the full list of included studies (cited as S1 and S2 Tables) is a significant omission; it was impossible to assess the included literature and assess it. Table S3 was missing as well.*

Our apologies for this omission. We have made sure that the three supplementary materials are now correctly attached.

*2. The manuscript includes broad assertions not always supported by the data. For example:
"Despite the increasing number of environmental health studies conducted in Canada among Indigenous populations, the elevated risk... is persistent and researchers are not fully communicating community-defined priorities in their publications."
I’ve interpreted this sentence as implying a causal link between persistent exposure and researchers’ failure to reflect community priorities, while generalizing about the entire field. Such claims overstate the evidence presented in a scoping review. It is important to acknowledge that academic publications are often constrained by journal formats, word limits, or editorial policies that may limit authors’ ability to include detailed community perspectives/priorities. Some researchers may, in fact, be aligning closely with community priorities in practice even if this is not fully reflected in published outputs. Claims throughout the manuscript should be more cautiously framed and more closely tied to the data presented to improve analytical rigour and credibility, or you run the risk of making generalized claims that are not necessarily true.*

Thanks. We agree that some of the assertions made could be more precise to avoid transmitting a message that does not align with the evidence or the arguments we aim to communicate. Indeed, the sentence you provided as an example can be interpreted in the way you exposed, which was not our intention. We reworded the manuscript as follows:

“While there is a relatively large and increasing number of environmental health studies conducted in Canada among Indigenous Peoples, the elevated risk of these populations to toxic chemical exposure is persistent.

One factor that may hinder the translation of study results into improving Indigenous environmental health in Canada is that researchers are not adequately including the unique perspective of Indigenous Peoples in their studies or, alternatively, they are not fully communicating community-defined priorities in their publications ^14,15^. To avoid cases in which researchers engage in meaningful collaborations with Indigenous Peoples but fail to report these partnerships in scientific publications, editorial policies should adapt to encourage explicit discussions on the role of community engagement in strengthening the rigour, effectiveness, and relevance of studies with a biomonitoring component.”

In addition, we agree with your concern that some researchers may closely engage with Indigenous Peoples even if this is not reflected in their publications. As authors, we have felt constrained and limited by journals in our ability to provide a broader and relevant context of the nature of our collaborations with Indigenous partners. In this manuscript, we discussed this issue in the *Beyond documenting damage: Community use of study results and Indigenous authorship subheading* and in the *Limitations and Strengths* section. Based on the concern you raised we expanded those discussions to further clarify our argument and the limitations of our study. The manuscript now reads as follows:

Third paragraph in *Beyond documenting damage: Community use of study results and Indigenous authorship* subheading:

“Although these results may, in fact, represent a concerning pattern in quantitative research, the limited discussion of community use of study results in research articles is likely partly associated with different scientific writing practices across research paradigms. Journals that publish qualitative research may expect and encourage researchers to report and discuss more broadly how the study was integrated into other social processes beyond the boundaries of the study design. This practice should be expanded and adapted to journals focused on quantitative studies, encouraging authors who are part of equitable partnerships with Indigenous Peoples to reflect and recognize the contribution of community engagement to the research and use of the study results.”

First Paragraph *Limitations and Strengths* section:

“Additionally, researchers who promote equitable partnerships with Indigenous Peoples may not be reporting on strategies to support community engagement and the use of the study results by the community in their scientific publications. Thus, our classification may not comprehensively reflect the characteristics of the research process, but the constraints established by editorial policies. Moreover, there could be cases where the limited information provided in articles regarding the role of the community in the research process is due to the community’s preference. However, in addition to the intrinsic value of evaluating researchers’ practices and discussions in scientific publications, we believe that our findings are indicative of general trends about the nature of current collaborations between environmental health researchers and Indigenous Peoples. Further research should be conducted to assess the quality of the community engagement activities and the use of study results beyond what is reported in scientific publications.”

Moreover, we identified additional arguments across the text that benefited from further clarification to avoid generalizations not supported by the results of this study. Below we noted the edited text in the new version of the manuscript.

*Bridging Western and Indigenous knowledge through community engagement* subheading (2^nd^ paragraph):

“In this review, however, we observed a relatively low proportion of articles reporting including Indigenous knowledge in the study design, and only two studies using Indigenous research methods in addition to Western approaches.”

*Community engagement strategies* subheading (2^nd^ paragraph):

“Although in some cases the lack of reporting could be caused by journals’ editorial constraints that limit authors’ ability to report existing community engagement activities, it may also be indicative of common concerning practices in environmental health research. The engagement of communities in the research process is particularly key to improving the sustainability and impact of studies that include a biomonitoring component ^54^.”

Country distribution of Indigenous food systems contamination research subheading (Last paragraph):

“One factor that may hinder the translation of study results into improving Indigenous environmental health in Canada is that researchers are not including the unique perspective of Indigenous Peoples in their studies or, alternatively, they are not fully communicating community-defined priorities in their publications ^14,15^. To prevent cases in which researchers engage in meaningful collaborations with Indigenous Peoples but fail to report these partnerships in scientific publications, editorial policies should adapt to encourage explicit discussions on the role of community engagement in strengthening the rigour, effectiveness, and relevance of studies with a biomonitoring component.”

*3. Only two studies are noted as using Indigenous research methods, but the manuscript	does not describe these approaches/ studies (this is mentioned 2-3 times). A brief	explanation would help clarify what qualifies as Indigenous research methodology in this	context.*

In the new version of the manuscript, we briefly described the two studies identified as using Indigenous research methods. This text was added to the third paragraph of the results:

“Briefly, Cott and colleagues collaborated with Indigenous experts to evaluate appearance-based traditional methods to determine food quality and contamination levels of fish consumed by local communities ^65^, while Lucier and collaborators applied storytelling methods to assess the multiple impacts of water quality degradation in an Anishinaabe First Nations community (Ontario, Canada) ^66^.”

Thus we added the following articles to the reference list:

Cott P, Amos A, Guzzo M, et al. Can traditional methods of selecting food accurately assess fish health? *Arctic Science*. 2018;4(2):205-222. doi:10.1139/as-2017-0052

Lucier KJ, Dickson-Anderson SE, Skead D, Skead K, Kosmas E, Schuster-Wallace CJ. ‘That water out there is no damn good for anybody’: Experiences with declining water quality in a First Nation community. *Can Water Resour J*. 2023;48(1):18-32. doi:10.1080/07011784.2022.2122084

In addition, we expanded the definition of Indigenous research methods provided in Table 3. The description now reads:

“Indigenous methods may be context-specific; however, they usually include strategies beyond data collection and are intended to build relationships and foster self-determination. For example, storytelling and talking circles.”

*4. Tables 2 and 3 contain a lot of useful data, but the main text mostly lists or repeats those numbers without interpreting what they mean or highlighting key patterns. The results section would benefit from clearer synthesis, such as drawing out trends, contrasts, or implications from the tables, rather than just presenting counts. This is one my biggest concerns, you have presented interesting findings, but you need to give the reader more of a “so what?” or “why should I care as a researcher, policy maker, community member, etc.?”*

We appreciate the opportunity to expand on the potential implications of our findings. We edited the results and discussion sections accordingly. In the revised version of the results section we added several clarifications to the descriptive presentation of the results, which are highlighted below.

*Results* section (3^rd^ paragraph) now reads:

“Studies were conducted in 22 countries from North America, South America, Eurasia, Oceania, and Africa, with five conducted across two countries. However, the vast majority of studies were located in North America. About half of the studies were conducted in Canada, followed by the United States, Brazil, Peru, and Russia. Only four studies were conducted in Africa or Asia (Table 2). Countries in the Americas are disproportionately represented in our sample, with no apparent correlation between the number of studies per country and their total Indigenous population. For example, no studies were conducted in Mexico and Guatemala, which have the largest number and proportion of Indigenous population in the Americas ^64^, while Canada accounted for more than half of all the studies included in the review. This suggests that more studies need to be conducted and published from countries with large Indigenous populations that are no doubt facing considerable environmental concerns.”

*Results s*ection (8^th^ paragraph) now reads:

“Approximately 60% of articles did not report on community use of study results for current or future projects, interventions, or programs (Table 3). When disaggregating by study methods, articles documenting the inclusion of a qualitative component or approaches including Indigenous knowledge reported a relatively higher proportion of current or expected strategies for the community to use the study results (Figure 3). The proportions of community results use categories were similar for studies using survey analysis, biological samples, or simulation approaches. The presence of community engagement activities is not exclusive to specific research methods. We identified the six community engagement categories across all research methods, except simulation or modelling methods, for which none of the studies reported to be requested by the community.”

*Results s*ection (9^th^ paragraph) now reads:

“In addition, we explored the potential associations between the absence or presence of authors with Indigenous affiliation, both with community engagement and community results use (Figures 4A and 4B). We identified mention of an Indigenous affiliation only in three countries: 37 in Canada, 17 in the United States, and one in Suriname. In the latter, the authors reported conducting an activity to receive community feedback and that the community was using the study results as part of an ongoing broader strategy. In Canada and the United States, studies with an Indigenous affiliation reported a higher proportion of community engagement activities than those without such affiliation. We also observed that studies having an author with an Indigenous affiliation reported a larger proportion of activities to receive community feedback than studies without an Indigenous affiliation. The proportion of studies requested by an Indigenous community was larger in studies with an Indigenous affiliation compared to those without. This was also the case for most community engagement strategies (i.e., collaboration in results discussion, collaboration in methods design, and community members conducting research activities). This could be indicative of an association between Indigenous authorship and community engagement in food systems contamination research.”

In addition, we expanded the discussion section to further discuss potential implications and actions in light of the findings described in the results section. Please find below the edits made to the discussion section:

Country distribution of Indigenous food systems contamination research subheading (2^nd^ paragraph):

“Furthermore, there is no single definition of Indigenous Peoples, as there are often differing views about the characteristics that define Indigeneity ^74,75^. This is further complicated by the ongoing impacts of colonization and the displacement of sovereignty, which continue to impact the social fabric of many Indigenous Peoples ^76^. Moreover, depending on the context, communities may choose not to self-identify as Indigenous due to political factors or, in some cases, as a strategy for survival. Thus, the social and political struggle of Indigenous Peoples cannot be separated from the scientific practice of environmental health research and the rigorous global assessment of contamination of Indigenous food systems.”

Country distribution of Indigenous food systems contamination research subheading (last paragraph):

“One factor that may hinder the translation of study results into improving Indigenous environmental health in Canada is that researchers are not including the unique perspective of Indigenous Peoples in their studies or, alternatively, they are not fully communicating community-defined priorities in their publications ^14,15^. To prevent cases in which researchers engage in meaningful collaborations with Indigenous Peoples but fail to report these partnerships in scientific publications, editorial policies should adapt to encourage explicit discussions on the role of community engagement in strengthening the rigour, effectiveness, and relevance of studies with a biomonitoring component.”

*Bridging Western and Indigenous knowledge through community engagement* subheading (3^rd^ paragraph):

“Despite the persistent challenges to articulate Indigenous and Western knowledge systems, fields like planetary health are progressively recognizing the key contributions of Indigenous knowledge to understanding the complex connection between humans and nature ^68^. As planetary health approaches are incrementally being included in higher education curricula, educators of health professionals and researchers should leverage this opportunity to promote innovative learning approaches that incorporate diverse knowledge systems ^86^. This could strengthen the relevance and effectiveness of future risk assessments and studies of Indigenous food systems. ”

New reference:

Brand G, Wise S, Bedi G, Kickett R. Embedding Indigenous knowledges and voices in planetary health education. The Lancet Planetary Health. 2023 Jan 1;7(1):e97–102.

*Community engagement strategies* subheading (4^th^ paragraph):

“Despite the potential benefits of community-based risk assessments, researchers and community members have reported persistent challenges in their implementation ^14^. Partnerships between academia and communities could benefit from reflecting on better strategies to leverage solutions to contamination issues ^91^. Previous studies have found that research directed by communities experiencing environmental injustice is more likely to result in action than research led by academic institutions ^91^. Thus, institutions of higher education should use their power and prestige to amplify communities’ voices, while expanding opportunities to play a supportive, rather than leading, role in environmental health research ^69,91^.”

New reference:

Davis LF, Ramírez-Andreotta MD. Participatory Research for Environmental Justice: A Critical Interpretive Synthesis. Environmental Health Perspectives. 2021 Feb;129(2):026001.

*Beyond documenting damage: Community use of study results and Indigenous authorship* subheading (2^nd^ to last paragraph):

“Indigenous Peoples have long advocated for self-determination and engagement with research conducted in their communities. They have been at the forefront of strategies to improve collaborations with non-Indigenous researchers. For example, the First Nations principles of ownership, control, access, and possession (OCAP^®^), were created as a guiding tool for Indigenous Peoples and researchers to support Indigenous information governance and data sovereignty ^97,98^. Thus, the scientific community must reciprocate these efforts and normalize conversations in scientific journals about community participation and the use of study results as part of broader acts of reconciliation with Indigenous Peoples ^93^. These conversations in academic journals could provide valuable insights into pressing issues such as the uncritical expansion of policies mandating data openness, without considering how they can create mistrust among Indigenous partners and further exclude these populations from participating in research ^99,100^.”

New references:

McCartney AM, Anderson J, Liggins L, Hudson ML, Anderson MZ, TeAika B, et al. Balancing openness with Indigenous data sovereignty: An opportunity to leave no one behind in the journey to sequence all of life. Proceedings of the National Academy of Sciences. 2022 Jan 25;119(4):e2115860119.

Walter M, Lovett R, Maher B, Williamson B, Prehn J, Bodkin-Andrews G, et al. Indigenous Data Sovereignty in the Era of Big Data and Open Data. Australian Journal of Social Issues. 2021;56(2):143–56.

***Editing suggestions***

*5. The writing is clear, but some sentences are quite long or dense, making them harder to follow than they need to be. You’re presenting the state of the art, be clear and concise!*

Thanks for your positive review and your suggestions to improve the readability of the paper. We carefully revised the manuscript, particularly the Introduction and Discussion sections, and simplified or split several sentences to make them easier to follow. We believe these changes are noticeable in the new version. As an example of how we considered your advice across the manuscript, these are two of the edits we made in the Introduction:

From:

“Pollution is one of the greatest threats to human health. It is the world’s leading environmental risk factor, claiming nine million deaths per year, triple the number of deaths caused by HIV/AIDS, malaria, and tuberculosis combined ^1–3^. Despite these alarming figures, there is considerable uncertainty regarding the multiple effects of chemical pollution exposure on human health, particularly among historically marginalized populations ^2–5^.”

To:

“Pollution is the world’s leading environmental risk factor, claiming nine million deaths per year, triple the number of deaths caused by HIV/AIDS, malaria, and tuberculosis combined ^1–3^. However, there is considerable uncertainty regarding the multiple effects of chemical pollution exposure on human health, particularly among historically marginalized populations ^2–5^.”

From:

“Researchers are increasingly including multi-level and system-oriented approaches to assess and identify structural solutions to the existential crisis of globalized pollution ^6^. This is the case for scholars working under the umbrella of the emergent planetary health field, which has seen rapid growth in the last decade ^7^. ”

To:

“Researchers are increasingly including multi-level and system-oriented approaches to assess and identify structural solutions to the globalized pollution crisis ^6^. This is the case for scholars involved in the emergent planetary health field, which has seen rapid growth in the last decade ^7^.”

In addition, we appreciate your specific suggestions to improve the readability of the manuscript. We have noted the edits made below each of your suggestions.

*Introduction
6. Line 76: “The growing complexity of environmental contamination, including new chemicals and mixtures and multi-level pollution drivers…”
Suggested: “…including emerging chemicals, mixtures, and multi-level drivers of pollution…”*

Now:

“The growing complexity of environmental contamination, including emerging chemicals, mixtures, and multi-level drivers of pollution, requires solutions beyond conventional risk management approaches based on source-by-source and pollutant-by-pollutant assessments.”

*7. Line 83: “This is the case of environmental health research in the Circumpolar North…”
Suggested: “This is particularly evident in environmental health research in the Circumpolar North…”*

Now:

“This is particularly evident in environmental health research in the Circumpolar North, where researchers may not fully recognize the changing political climate in the region where research is viewed as an act of self-determination to promote Indigenous populations well-being^15^”

*8. Line 104: “Unfortunately, despite the increasing interest in fostering collaborations with Indigenous Peoples…”
Suggested: “Although interest in fostering collaboration is increasing…”*

Now:

“Although interest in fostering meaningful collaborations with Indigenous Peoples is increasing, Indigenous Peoples continue to experience greater disease burdens in comparison to the general population^17-19^.*”*

*Methods
9. Line 178: “Cited or citing references were not examined, and additional studies were not sought through other strategies.”
Suggested: “We did not examine cited or citing references, nor did we search grey literature or consult external sources.”*

Thanks, we edited the text accordingly.

*10. Line 196: “We verified if organizations were governed by Indigenous Peoples and classified those that explicitly mention their connection…”
Suggested: “We verified whether the organizations were governed by Indigenous Peoples…”*

Now:

“We verified whether the organizations were governed by	Indigenous Peoples and classified those that explicitly mention their connection to an Indigenous Nation as “Indigenous affiliation”.”

*Results
11. Line 220: “This number was based on the names that authors assigned in their	articles…” [I found this sentence a bit vague, starting on line 219.]
Suggested: “This count reflects the names used by authors in the original studies…”*

Thanks, we edited this line following your suggestion.

*12. Line 252: “We also explored the activities that were reported to have been carried	out…” Suggested: “We also examined reported community engagement activities…”*

Now:

“We also examined reported community engagement activities	and information on community use of the study results.”

*13. Line 281: “Around 60% of articles did not provide information on community use of	study results…” Suggested: “Approximately 60% of articles did not report…”*

Now:

“Approximately 60% of articles did not report on community use of study results for current or future projects, interventions, or programs (Table 3). ”

*Discussion
14. Line 365: “Despite the challenges of articulating contrasting knowledge systems…”
Suggested: “Despite the challenges of bridging Indigenous and Western knowledge systems…”*

Now:

“Despite the challenges of bridging Indigenous	and Western knowledge systems and building relationships with community members^14,76^,	there is mounting evidence of the benefits of these collaborative efforts on	addressing environmental justice issues^49,51,54^.”

*15. Line 431: “Community engagement strategies are not exclusive to qualitative methods…”
Suggested: “Although not exclusive to qualitative methods, community engagement strategies were more commonly reported in studies that included them.”*

Now:

“Although not exclusive to qualitative methods, community engagement strategies were more commonly reported in studies that included them. ”

*Conclusion
16. Line 565: “Beyond the benefits of a horizontal dialogue in incorporating analytical frameworks…”
Suggested: “Beyond the benefits of more equitable collaboration and integrated approaches…”*

This is the new version:

“Beyond the benefits of more equitable collaboration and integrated approaches that better explain the complexity of toxic exposures in Indigenous Peoples, co-developing interventions and research projects to evaluate and manage risk while considering nutritional benefits and cultural importance of traditional foods could constitute an act toward reconciliation itself.”

*17. Line 571: “Research on toxic exposures among historically oppressed populations needs to be in line with the voices of Indigenous scholars…”
Suggested: “Research on toxic exposures in historically marginalized populations should align with the priorities and perspectives of Indigenous scholars…”*

Thanks. The sentence now reads:

“Research on toxic exposures in historically oppressed populations should align with the voices of Indigenous scholars advocating to reframe environmental health research to also capture the hope, wisdom and stewardship of communities.”

*18. Finally, it would help to standardize terminology, for example terms like “Indigenous Peoples” and “Indigenous populations” and/ or “Indigenous communities” seem to be used interchangeably, and a consistent choice would improve clarity.*

Thanks for the suggestion. We agree that the article would benefit from standardizing. We changed “Indigenous populations” and “Indigenous communities” to “Indigenous Peoples” across the text where appropriate.

*With these revisions, the manuscript will be well-positioned to make a meaningful and	lasting contributions to the literature on Indigenous health, environmental contamination,	and improve research relationships with Indigenous partners.*

We appreciate your positive review, feedback and comments.

**Reviewer #2**

*1. Thank you for your work in this area. A comprehensive systematic search of the literature following a scoping review process was conducted to identify and describe reported practices for engaging Indigenous communities in research focused on contaminated food systems. The manuscript is well-written but would benefit from a few revisions to strengthen the methodological approach described and the implications of the scoping review findings.*

Thanks for your positive review and suggestions to strengthen the manuscript.

2. *Currently, the method section is described at a high level. The authors may consider providing further details on their search strategy, eligibility criteria, data extraction, classification, and analysis. Additionally, the authors may consider citing the process they followed for the systematic scoping review. Providing details on how articles were screened and selected for data extraction would help to highlight the systematic steps the research team took to ensure rigour and reduce bias in the scoping review process.*

We revised the methodology section and decided to reiterate and expand in the main text on some of the details included in Table 1 (“Scoping review search strategy and exclusion criteria used to select publications”).

The first paragraph of the methods section now reads:

“The search was limited to research articles on the contamination of Indigenous food systems that included a human health dimension in the data collection or analyses. Our research strategy aimed to capture a broad group of Indigenous food systems, Indigenous Peoples, and methodologies used to assess the potential association between food contamination and human health (Table 1). Thus, we did not restrict the type of methods used, food sources analyzed, study location, or the language of the study. To be included in the review, research articles needed to empirically assess chemical contamination, directly or indirectly, and discuss potential or measured impacts on human health or well-being. Moreover, included studies had to assess contamination of Indigenous food systems, and be conducted in an Indigenous territory or among Indigenous Peoples. We searched for research articles between January 2010 and July 29, 2024 (the day when the search concluded) in three databases, each containing several collections (Web of Science, Scopus, CAB direct and global). We did not examine cited or citing references, nor did we search grey literature or consult external sources.”

The second paragraph of the methods section now reads:

“The search process and results were documented following the Preferred Reporting Items for Systematic Reviews (PRISMA) approach ^58-60^. To identify studies that met our inclusion criteria we screened the articles in two phases. First, we screened the title and abstract and retained articles that met the inclusion criteria and articles that presented insufficient information at this stage to determine eligibility. Second, we conducted a full-text screening of the articles retained in the previous phase. We then reviewed the full manuscripts of the retained articles and extracted the data using a structured data collection form developed by the authors. Each article was screened and reviewed independently by two co-authors, and disagreements in the data extracted were resolved by consensus. To promote consistency, the first author screened and reviewed all the articles, while HK screened and reviewed articles published between 2010 and 2022 and KM between 2023 and 2024. We used the Covidence web-based collaboration software platform to identify duplicates, screen articles, identify conflicts to be resolved, develop the data extraction form, and extract data^61^.”

*3. Lines 169-171 read as an objective statement that could be moved to the introduction.	Your methods section could then lead with a description of the search strategy.*

We agree and moved these lines to the Introduction. The last sentence of the Introduction now reads:

“We contribute to filling this gap by conducting a systematic scoping review of empirical research articles that study contamination of Indigenous traditional food systems. This review aims to identify to what extent researchers report both the engagement of communities in the research process and how communities make use of study results.”

4. *Inclusion/exclusion criteria – Providing further information on your inclusion criteria,	including geographic context, type of source (could be more specific to indicate primary	research?), would help to clarify the scope of the review to the reader and set up how your	results are currently organized and presented.*

Thanks for the suggestion. We addressed this issue in your comment number 2.

*5. Data analysis – more information is needed here. The authors indicate using R	statistical software, but don’t describe what it was used for and why.*

We expanded the text accordingly. It now reads:

“Data cleaning, calculation of descriptive statistics and proportions, and graphs were conducted in the R statistical computing environment (version 4.1.1), and the code was executed in RStudio (version 1.4.1717) ^62,63^. ”

*6. Information on the role of co-authors in either developing the search strategy,	eligibility criteria, screening and article selection could be provided. For example, in	lines 190-191, you could include the initials of the two independent reviewers.*

We appreciate your comment and edited the text accordingly:

“To promote consistency, the first author screened and reviewed all the articles, while HK screened and reviewed articles published between 2010 and 2022 and KM between 2023 and 2024.”

Additional information on the role of each author was provided in the article’s submission using the CRedit taxonomy. This information would be available in the published version of the article.

*7. As the authors indicate that their study as a scoping review, I suggest the authors check	their paper against the PRISMA Extension for Scoping Reviews (PRISMA-ScR): Tricco,	A. C., Lillie, E., Zarin, W., O'Brien, K. K., Colquhoun, H., Levac, D., Moher, D., Peters,	M. D. J., Horsley, T., Weeks, L., Hempel, S., Akl, E. A., Chang, C., McGowan, J., Stewart,	L., Hartling, L., Aldcroft, A., Wilson, M. G., Garritty, C., Lewin, S., … Straus, S. E.	(2018). PRISMA Extension for Scoping Reviews (PRISMA-ScR): Checklist and	Explanation. Annals of internal medicine, 169(7), 467–473.* [*https://doi.org/10.7326/M18-*](https://doi.org/10.7326/M18-)

Thanks for suggesting this key reference. As part of the PLOS ONE requirements, we submitted a PRISMA-ScR Checklist. We should have added this reference to the methodology section and it is now included in the revised version of the manuscript. In addition, we revisited the Tricco and colleagues’ article to inform the edits elaborated under your previous comments. The following reference is now included in the reference list:

Tricco AC, Lillie E, Zarin W, et al. PRISMA Extension for Scoping Reviews (PRISMA-ScR): Checklist and Explanation. *Ann Intern Med*. 2018;169(7):467-473. doi:10.7326/M18-0850
